# Systematic design of DMBT1-derived peptides correlating physicochemical properties and sequence motifs with siRNA delivery and efficacy in cancer therapy

**DOI:** 10.1016/j.ebiom.2025.105977

**Published:** 2025-10-22

**Authors:** Martina Tuttolomondo, Mikkel Green Terp, Nazmie Kalisi, Stefan Vogel, Henrik Jørn Ditzel

**Affiliations:** a Department of Molecular Medicine, Unit of Cancer Research, University of Southern Denmark, 5000, Odense, Denmark; b Department of Physics, Chemistry and Pharmacy, University of Southern Denmark, 5000, Odense, Denmark; c Department of Clinical Research, University of Southern Denmark, 5000, Odense, Denmark; d Department of Oncology, Odense University Hospital, 5000, Odense, Denmark

**Keywords:** Cell-penetrating peptides, siRNA, RNA interference, Cancer therapy, DMBT1

## Abstract

**Background:**

Molecules driving the cancer process are frequently difficult to target with traditional small-molecule drugs. Small interfering RNAs (siRNAs) offer high specificity, but their clinical translation is hindered by inefficient delivery and rapid degradation. We previously identified DMBT1-derived cell-penetrating peptides (CPPs) that encapsulate siRNA and improve serum stability in vitro.

**Methods:**

We designed 37 DMBT1-derived peptides using a rational, high-throughput pipeline to enhance siRNA encapsulation, stability, and delivery. Binding, uptake, and silencing were assessed in A375 and MCF7 cells. Regression and motif discovery analyses were applied to link peptide physicochemical features with encapsulation efficiency, serum stability, and gene silencing.

**Findings:**

Twenty-seven peptides showed improved siRNA binding and 20 achieved robust uptake in serum. We identified a conserved motif, SWGRVRVLRGDKW, enriched in complexes achieving >75% knockdown, associated with efficient cytosolic release. HE25 emerged as the lead peptide, delivering BRAF^V600E^-siRNA and significantly reducing A375 proliferation in vitro. In female NOG CIEA mice xenografts, HE25 suppressed tumour growth, while repeated intravenous dosing in BALB/c mice confirmed biosafety.

**Interpretation:**

Targeted optimisation combined with motif-based design establishes a framework for developing next-generation CPPs. The identification of a conserved motif driving efficient delivery highlights new opportunities for advancing siRNA therapeutics in cancer and beyond.

**Funding:**

This work was supported by 10.13039/501100009708Novo Nordisk Foundation, 10.13039/100008398Villum Foundation, 10.13039/501100003554Lundbeck Foundation, 10.13039/501100005747A.P. Møller Foundation, 10.13039/100007403Dagmar Marshalls Foundation, Neye Foundation, 10.13039/501100010347Fabrikant Einar Willumsens Mindelegat, and Direktør Michael Hermann Nielsens Mindelegat.

**Research in context**

**Evidence before this study**

Effective clinical application of siRNA therapeutics has been hampered by insufficient *in vivo* delivery and rapid degradation in serum. While a variety of lipid nanoparticles and natural or synthetic cell-penetrating peptides (CPPs) have been explored, most suffer from suboptimal encapsulation efficiency, limited serum stability, and poor endosomal escape. In our prior work, we reported DMBT1-derived CPPs capable of siRNA encapsulation and in vitro stability; however, these early constructs failed to achieve robust gene knock-down when tested under serum-supplemented conditions (2.5% FBS), which were used as a standardised in vitro challenge to assess peptide-mediated siRNA protection.

**Added value of this study**

Here, we present a systematic design and evaluation of 37 DMBT1-derived peptides optimised for siRNA binding, serum stability, and functional delivery. Through comprehensive correlation analyses and motif discovery, we identified the conserved sequence SWGRVRVLRGDKW as a key determinant of efficient intracellular trafficking and siRNA release. Among our candidates, the peptide HE25 emerged as a potent siRNA carrier, demonstrating effective BRAF^V600E^-targeted gene silencing in A375 melanoma cells in vitro and significant tumour growth suppression in xenograft models.

**Implications of all the available evidence**

By linking physicochemical properties and specific sequence motifs to delivery performance, this work establishes a rational framework for next-generation CPP development. Our findings not only advance mechanistic understanding of peptide-mediated siRNA transport but also pave the way for translational evaluation of HE25 and related peptides as clinically viable siRNA delivery vehicles in precision oncology and beyond.

## Introduction

RNA interference (RNAi) is a powerful and promising therapeutic approach for selectively and reversibly silencing target genes. siRNA therapeutics have received recent FDA approval for treating various diseases, including amyloidosis, acute hepatic porphyria and polyneuropathy.^1^ This technology can potentially address other severe diseases like cancer by targeting undruggable oncogenes, overcoming resistance and enhancing precision for personalised therapies.^2^

Despite advancements in siRNA therapeutics through chemical modifications and delivery systems, challenges like efficient targeting, systemic stability and controlled intracellular release still limit their full therapeutic potential.^3^, ^4^, ^5^, ^6^ Lipid nanoparticle (LNP) systems have revolutionised nucleic acid delivery, as seen in mRNA vaccines, and offer promise for siRNA-based cancer therapies. However, challenges like immunogenicity, batch variability and limited tumour targeting hinder their application in cancer treatment. These limitations highlight the need for alternative or complementary delivery strategies to enhance therapeutic efficacy in siRNA-based cancer treatment.^7^^,^^8^

Cell-penetrating peptides (CPPs) offer a promising approach to address these limitations.^9^, ^10^, ^11^ These short peptides, capable of traversing cellular membranes, can facilitate delivery of therapeutic molecules like siRNAs using two different binding techniques, covalent conjugation and non-covalent complexation. Covalent conjugation involves chemically linking the siRNA to the peptide, enhancing stability and delivery precision,^12^ while non-covalent complexation forms stable complexes that protect siRNA from enzymatic degradation and facilitate efficient intracellular release.^13^^,^^14^

Non-covalent complexation has particularly demonstrated success *in vivo*, forming stable yet flexible complexes that ensure siRNA functionality while minimising issues such as rapid clearance or toxicity.^15^ This process relies on electrostatic interactions between the positively-charged residues of CPPs and the negatively-charged siRNA backbone, along with additional non-covalent forces such as hydrogen bonds, π-stacking and van der Waals forces. Together, these forces ensure robust encapsulation, enhancing stability and delivery efficiency in physiological environments.^16^, ^17^, ^18^ By forming a protective peptide shell around the siRNA, this method not only enhances stability in biological fluids and extends the siRNA half-life in the circulation but also allows for precise control over the size and charge of CPP–siRNA complexes, optimising delivery to various target tissues including tumours.^19^ Additional advantages of non-covalent complexation of siRNA with CPPs include low cost, ease of formulation, and high tunability.^9^

Beyond their delivery mechanisms, CPPs provide the unique advantage of multifunctionality over other carriers like nanoparticles and lipid-based systems. Their versatility enables the incorporation of several sequences within a single peptide, allowing them to perform multiple simultaneous tasks such as tumour targeting, endosomal escape and siRNA stabilisation.^20^, ^21^, ^22^

Although CPPs offer significant advantages, their widespread application is still constrained by challenges such as cytotoxicity and immunogenicity, particularly in *de-novo* designed peptides.^23^ Literature suggests that protein-derived CPPs of mammalian origin often exhibit superior biocompatibility and lower immunogenicity, making them safer for *in vivo* applications.^24^ This may be particularly relevant for CPP/siRNA complexes with low or near-neutral surface charge, which are expected to exhibit fewer nonspecific interactions with serum proteins and immune components.

Among human proteins suitable for CPP design, Deleted in Malignant Brain Tumours 1 (DMBT1), a pivotal molecule in the innate immune system known for its interaction with nucleic acids, has served as a valuable inspiration for the development of human-derived CPPs.^25^, ^26^, ^27^

We have identified two human DMBT1-derived peptides, SRCRP2-11 and SRCRP2-11R, demonstrating self- and co-assembly behaviour with siRNA, enabling siRNA delivery *in vitro.*^28^ These peptides exhibited stabilisation of siRNA through electrostatic and additional non-covalent interactions; however, their limited biological effects in serum-containing environments highlighted the need for improved serum stability in CPP designs.^28^

Despite promising preclinical results, the clinical translation of most CPPs has been hindered by a lack of rational design strategies.^29^^,^^30^ Building on our discovery of DMBT1-derived peptides, we designed peptide variants with enhanced cell penetration, self-assembly and serum stability for siRNA delivery. Using systematic sequence modifications and advanced analyses, we linked physicochemical properties to key outcomes like siRNA binding, stability and gene silencing. This study introduces a systematic approach to CPP design, addressing limitations through correlation analyses and experimental validation.

In this study, we asked whether systematic rational design of DMBT1-derived CPPs could improve the delivery of siRNA in serum-containing conditions and achieve therapeutic efficacy in cancer models. Our objective was to generate and test a library of CPP variants, linking sequence features and physicochemical properties to siRNA encapsulation, stability, uptake, and gene silencing. We hypothesised that specific sequence motifs and optimised charge/hydrophobic balance would enhance endosomal escape and cytosolic release, thereby improving in vitro and *in vivo* gene silencing efficacy.

Among the DMBT1-derived peptides developed (DCPPs) using this method, HE25, previously optimised for RdRP silencing in SARS-CoV-2, has demonstrated effective siRNA delivery to cancer cells.^31^ In this study, HE25 achieved robust gene silencing and significantly reduced cancer cell proliferation, even in serum-containing media, *in vitro* and *in vivo*, underscoring its potential for therapeutic applications in cancer.

This study offers insights that may guide the development of highly effective CPP-based siRNA delivery systems for broad therapeutic applications.

## Methods

### Peptides and siRNAs

Peptides (>95% purity; GenScript) were dissolved at 1 mM in RNase-free water—or, for poorly soluble sequences, first in DMSO then diluted to 1% DMSO in water. EMSA employed Thermo Fisher's Stealth RNAi™ Negative Control siRNA. Serum-stability assays used GenScript HPLC-purified FRET siRNA duplexes (sense: FAM-5′-GGUCUAGCUACAGAGAAAU-3′-TAMRA; antisense: FAM-5′-AUUUCUCUGUAGCUAGACC-3′-TAMRA). Cell uptake studies used BLOCK-iT™ Alexa Fluor™ Red control, GAPDH knock-down used Silencer GAPDH siRNA, and BRAF^V600E^ targeting used custom Stealth siRNA (GGUCUAGCUACAGAGAAAUCUCGAU).

Unless otherwise specified, negative controls consisted of PBS, peptide alone or non-targeting control siRNA/HE25 complexes.

### Electrophoretic mobility shift assay

Peptides (10–100 μM) were mixed with siRNA at 1:10–1:100 molar ratios in RNase-free water and incubated 30 min at 37 °C (or 1 h at 50 °C for low-binding peptides). Complexes, supplemented with 5% glycerol, were run on 4% agarose gels in MOPS buffer (pH 7.5) at 70 V for 30 min, stained with GelRed, and imaged under UV. FastRuler UltraLow served as the size marker.

### Serum stability assay

DCPP/FRET siRNA complexes were prepared at their respective binding molar ratios, following the protocol described for the electrophoretic mobility shift assay. The complexes were then incubated with 2.5% FBS diluted in PBS for 30 min at 37 °C to assess stability. Following incubation, proteinase K (28 μg/mL) was added to inhibit serum RNases. To disassemble the complexes and release the FRET-labelled siRNA, the DCPP/FRET siRNA mixtures were treated with dextran sulphate sodium (DSS, 2.8 mg/mL), urea (0.22 mg/mL) and SDS (0.28%). Samples, including FBS-treated or untreated FRET-siRNA as well as FBS-treated or untreated DCPP/FRET siRNA complexes (assembled or disassembled), were mixed with dye-free loading buffer (final glycerol concentration of 5%) and subjected to electrophoresis on a 4% (w/v) agarose gel without staining. Electrophoresis was conducted under the same conditions as described for the electrophoretic mobility shift assay. Gels were imaged using the Spectrum *In vivo* Imaging System (IVIS, PerkinElmer) with excitation at 465 nm and detection at 520 nm for FAM and 600 nm for TAMRA, with an exposure time of 15 s. Image analysis was performed using the FIJI ImageJ gel analyser tool (NIH). FRET efficiency was calculated as described in our previous work.^32^^,^^33^

### Cell internalisation experiments

A375 (RRID: CVCL_0132) and MCF7 cells (RRID: CVCL_0031) were obtained from ATCC. MEL-STV cells (here called MelST, RRID: CVCL_9U71) are immortalised human melanocytes generated by introducing SV40 large T antigen and hTERT. They are non-tumorigenic and were kindly provided by Dr. Robert Allan Weinberg (Whitehead Institute, MIT). All cell lines were cultured in DMEM + GlutaMax™ with 5% FBS at 37 °C/5% CO_2_. Working solutions of Alexa Fluor 555 siRNA were prepared in UltraPure DNase/RNase-Free Distilled Water at concentrations of 100–400 nM, and peptide solutions were prepared to achieve the desired peptide–siRNA binding molar ratios, selected based on the siRNA binding efficiency of each peptide. siRNA was mixed with its respective peptide solution and incubated for 30 min at 37 °C or 1 h at 50 °C, depending on the peptide. After incubation, the complexes were diluted in OptiMEM and added to the wells at 50 μL per well, with final siRNA concentrations ranging from 25 to 100 nM. A375 and MCF7 cells were seeded at a density of 2000 cells per well in 96-well plates. Fifty microlitres of the cell suspension was added directly onto the pre-formed siRNA–peptide complexes, resulting in a final medium volume of 100 μL per well. Cell suspensions were prepared in DMEM, with or without FBS, adjusted to a final concentration of 2.5%, depending on the experimental conditions. After 24 h of incubation, the media were removed, and cells were washed with PBS. OptiMEM without phenol red was added, and fluorescence was measured using Alexa Fluor 555 (excitation/emission: 553/568 nm, reading: 535/595 nm). Cells were subsequently stained with crystal violet and resuspended in citrate buffer, and absorbance was measured. Crystal violet absorbance was used to normalise the fluorescence signal, accounting for cell density or viability differences.

### Nanoparticle tracking analysis and zeta potential

Particle size, concentration, and zeta potential were analysed using a ZetaView Nanoparticle Tracking Analyzer PMX-220 (Particle Metrix, Germany). BRAF^V600E^ siRNA was encapsulated with HE25, SREP4R, HR25, TDHR, HCRRGD, or CHP2R peptides at their respective binding molar ratios in ultrapure water to a final volume of 1 ml. Samples were diluted to achieve an optimal concentration of 140–200 particles/frame. Measurements were recorded at 10 cell positions under the following capture settings: temperature, 23–25 °C; sequence length, 30; frame rate, 30 fps; switch frame, 10; video resolution, medium; laser wavelength, 488 nm; sensitivity, 60–80; and shutter, 100–200. Analysis parameters were set to: minimum size, 1.0 nm; maximum size, 1000 nm; trace length, 12; and tracking radius, 3 pixels. For each condition, three independent stocks were prepared. Size measurements were performed in triplicate, while zeta potential was measured in duplicate, with 100 μL of fresh sample injected prior to each measurement. Data were processed using ZetaView software (PEX).

### Aggregation assay

Peptides (HE25, SREP4R, HR25, TDHR, HCRRGD, CHP2R; 0–10 μM) were incubated in ultrapure water in the absence or presence of BRAF^V600E^ siRNA (0.1 μM final in well) at 37 °C for 30 min or 50 °C for 1 h (siRNA/HE25), followed by equilibration to room temperature. Complexes were transferred to 96-well black plates (80 μL per well) and supplemented with 20 μL of Bis-ANS (5 μM), Nile Red (1 μM), or Thioflavin T (10 μM). Controls without dye received water. After incubation for 30 min at room temperature with gentle shaking, absorbance at 340 nm and fluorescence (Bis-ANS: ex400/em480; Nile Red: ex550/em630; ThT: ex440/em485) were measured on an i3X plate reader (top-read mode). Two independent experiments (n = 2) were performed.

### Transfection and silencing experiments *in vitro*

GAPDH silencing: DCPP/GAPDH-siRNA complexes were prepared as above, diluted in OptiMEM, and added (50 μL) to 96-well plates. A375 and MCF7 cells (2 × 10^3^ cells/well) in 50 μL OptiMEM + 5% FBS + 1% P/S were plated onto the complexes (final 100 μL, 2.5% FBS). After 48 h, GAPDH knock-down was measured with the KDalert™ kit.

**Fig. 9:**
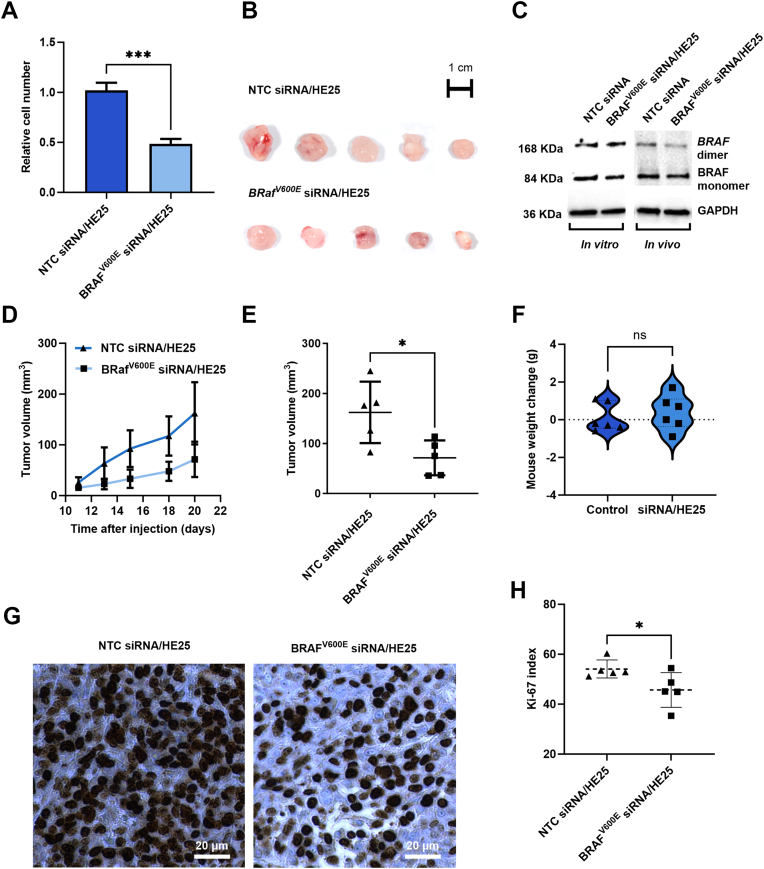
**BRAF^V600E^ silencing in melanoma *in vitro* and *in vivo* using cell-penetrating peptide HE25/BRAF^V600E^ siRNA complexes**. (A) *In vitro* silencing of BRAF^V600E^ in A375 melanoma cells, showing decreased cell proliferation after treatment with BRAF^V600E^ siRNA complexed with HE25. (B) *In vivo* inhibition of tumour growth by BRAF^V600E^ siRNA/HE25 complexes in A375 xenograft models, with representative images of tumours collected at the 20-day endpoint (C) Western blot analysis of BRAF^V600E^ protein expression following BRAF^V600E^ siRNA treatment in vitro and *in vivo*, with corresponding band quantification presented in Supplementary Figure S7. (D) Tumour growth curves over 20 days and (E) endpoint tumour volumes corresponding to the images shown in (B). (F) Mouse body weight changes after 15 days of treatment with siRNA/HE25. (G) Representative Ki-67 immunostaining of tumours from mice treated with BRAF^V600E^ siRNA/HE25 or NTC siRNA/HE25 corresponding to the images shown in panel B. (H) Quantification of Ki-67 staining in tumours corresponding to images shown in (B) (n = 5). Data were analysed using https://www.ihcexpert.com and GraphPad Prism, and statistical significance was determined by Student's t-test (∗P < 0.05; ∗∗P < 0.01; ∗∗∗P < 0.001; ∗∗∗∗P < 0.0001).

BRAF^V600E^ silencing: BRAF^V600E^ siRNA/HE25 complexes were prepared by mixing HE25 peptide and BRAF^V600E^ siRNA solutions at the indicated molar ratios, incubating for 1 h at 50 °C, and diluting in OptiMEM (50 μL/well). Cells were seeded in 50 μL medium supplemented with 5% FBS and 1% P/S on top of the complexes (final 100 μL, 2.5% FBS). For the experiment in Fig. 9A, A375 cells (1 × 10^3^/well) were transfected with HE25/siRNA complexes (1:100, 20 nM siRNA) and cell viability was assessed after 4 days by crystal violet staining. For the experiment in Supplementary Figure S7, A375 parental (1 × 10^3^/well) and MelST melanocytes (4 × 10^3^/well) were transfected with HE25/siRNA complexes (1:80, 50 nM siRNA), and cell viability was measured after 3–4 days using the CellTiter-Blue assay (Promega) according to the manufacturer's instructions.

### Live-cell endosomal tracking and lysosomal colocalisation

A375 melanoma cells were transduced with BacMam GFP 2.0 early endosome reagent (Thermo Fisher Scientific) according to the manufacturer's instructions and maintained in DMEM supplemented with 5% FBS and 1% penicillin/streptomycin. For transfection assays, cells were seeded at 2 × 10^3^ cells/well in 96-well ImageLock plates (Sartorius). Alexa Fluor 555-labelled siRNA (50 nM final concentration, Block-iT, Thermo Fisher Scientific) was complexed with peptides (HE25, SREP4R, HR25, TDHR, HCRRGD, CHP2R) at molar ratios ranging from 1:10 to 1:100. Complexes were prepared by incubating peptides (0.5 mM stock, diluted in ultrapure water) with siRNA for 1 h at 50 °C (HE25) or 30 min at 37 °C (all others), followed by dilution in OptiMEM (5% FBS, no phenol red). Cells were then resuspended in pre-equilibrated OptiMEM and added to the peptide–siRNA complexes (192 μL cell suspension per condition), incubated for 30 min at 37 °C, washed, and reseeded in fresh OptiMEM (2.5% FBS). Live-cell imaging was performed every 30 min for 24 h using an IncuCyte system (Sartorius) in red (AF555-siRNA), green (GFP-endosomes), and phase contrast channels at 20× magnification.

For immunofluorescence, cells were fixed 24 h post transfection in 10% formalin, permeabilised with 0.1% Triton X-100, and stained with HCS CellMask™ (Thermo Fisher Scientific). After blocking with 10% normal goat serum, cells were incubated with anti–LAMP-1 monoclonal antibody (Transduction Laboratories, 611042, RRID: AB_398355; 1:100) overnight at 4 °C, followed by Alexa Fluor 350–conjugated goat anti-mouse secondary antibody (Invitrogen, A21049; 1:250, RRID: AB_2535717). Cells were washed and imaged with an APX1000 fluorescent microscope at 20× magnification.

For the live-cell endosomal experiment, colocalisation between green and red fluorescence channels was quantified for each sample and time point using a custom Python script (Python v3.10). For each image pair, green and red TIFF files were imported with scikit-image and converted to floating-point arrays. Pixel intensities from the entire image were flattened into vectors and Pearson's correlation coefficient was computed using the scipy.stats.pearsonr function. Results for all image pairs were compiled into a CSV file for further analysis.

For the immunofluorescence experiment with lysosomal staining, colocalisation between green and red fluorescence channels was quantified in ImageJ by calculating Pearson's and Mander's correlation coefficients.

### *In vivo* experiments

For the BRAF^V600E^ silencing experiments *in vivo*, 7-week-old female NOG CIEA mice (average body weight ∼20 g, Taconic, Denmark) were kept under SPF conditions. Mice were randomised into treatment groups based on body weight at the start of the study. Randomisation by tumour size was not feasible because treatments commenced immediately together with xenograft injection. Investigators were not blinded during group allocation or outcome assessment. HE25 was complexed with BRAF^V600E^ or control siRNA (13 pmol), incubated at 50 °C for 1 h, then mixed with 2.5 × 10^5^ A375 cells in DMEM (pH 6.8) and injected subcutaneously (n = 5 per group). Tumour size was tracked by calliper for 14 days (volume = width^2^ × length/2), then tumours were bisected, with half fixed in formalin for histology and half snap-frozen at −80 °C for molecular analyses. Ki-67 staining of tumour sections was performed by the Immunohistochemistry Core Facility, Department of Pathology at Odense University Hospital, using standard immunohistochemistry protocols. Images were acquired with an EVOS microscope at 20× magnification, and Ki-67–positive cells were quantified using the ihcexpert.com Ki-67 quantification tool.

For the biosafety studies, female BALB/c mice (average body weight ∼20 g, Taconic, Denmark) were used. The siRNA/HE25 complexes were prepared at a molar ratio of 1:100, following the protocol outlined above, and administered via intravenous injection at a dosage of 0.2 mg/kg. This treatment regimen was repeated biweekly for a total of 15 days. Mice were weighed every 5 days throughout the 15-day period to monitor their health and response.

### Protein extraction and western blotting

For the in vitro BRAF^V600E^ silencing experiments, cells were transfected as described above. At the endpoint, protein was extracted in RIPA buffer supplemented with protease and phosphatase inhibitors, and total protein concentration was determined using the Pierce BCA Protein Assay Kit (Thermo Fisher Scientific) according to the manufacturer's instructions.

For the *in vivo* experiments, tumours were homogenised in RIPA buffer with protease/phosphatase inhibitors (2 × 20 s at 550 rpm), centrifuged at 4 °C, and supernatants collected. Protein content was quantified by BCA assay (37 °C, 30 min; absorbance at 562 nm).

For Western blot analysis, lysates were mixed with RIPA sample buffer (containing DTT and urea), boiled for 5 min, and alkylated with iodoacetamide. Samples and molecular weight markers were resolved on SDS-PAGE gels (200 V, 30 min) and transferred to PVDF membranes. After blocking with 0.5% TBST/5% milk, membranes were incubated overnight at 4 °C with 1 μg/mL rabbit polyclonal anti–B-Raf antibody (Santa Cruz, SC-166, RRID: AB_630938), followed by incubation with HRP-conjugated secondary antibody (Dako, P0448, RRID: AB_2617138) and detection by enhanced chemiluminescence (ECL—ThermoScientific, 34580). Membranes were subsequently stripped and re-probed with anti-GAPDH and HRP secondary antibody (Santa Cruz, sc-32233, RRID: AB_627679) to confirm equal protein loading. Membranes were imaged using ChemiDoc MP (Bio-Rad). Densitometric quantification of Western blot bands was performed using the Gel Analysis function in ImageJ.

### Ethics

All animal experiments were performed at the animal core facility at the University of Southern Denmark and approved by the Animal Experiments Inspectorate of the Ministry of Food, Agriculture and Fisheries of Denmark (Licence no. 2021-15-0201-00843). All procedures were performed in compliance with the ARRIVE guidelines and relevant national legislation on animal welfare. All mice were purchased from Taconic (Denmark) and maintained under specific pathogen-free (SPF) conditions. Female NOG CIEA mice are severely immunodeficient (Prkdc^scid^, Il2rg^null^) and were housed in a separate area under special precautions. Female BALB/c mice (∼20 g) are immunocompetent and non-genetically modified. Males were not used to avoid aggressiveness and stress from fighting. No animals had undergone previous procedures before the start of the experiments.

### Animal housing and husbandry

Mice were housed in groups of five in individually ventilated cages (IVCs) with autoclaved bedding, nesting material, and shelters provided as environmental enrichment, under a 12 h light/dark cycle (lights on 6 a.m. to 6 p.m.) at 21 ± 1 °C and 40–60% relative humidity, with ad libitum access to standard rodent chow and water. Animals were acclimatised for 2 weeks before initiation of experiments.

### Welfare and monitoring

To reduce pain, suffering, and distress, all procedures were performed by trained personnel following approved protocols. No expected or unexpected adverse events were observed during the experiments. Humane endpoints were predefined: mice were monitored every two days for signs of pain or discomfort, and animals were euthanised if tumours reached a maximum length of 0.8 cm, as required by the approved licence.

### Statistics

No formal study protocol was registered prior to the start of this work. The research question, design features, and analysis plan were developed internally and documented in laboratory notebooks but were not preregistered in a public database.

For in vitro experiments, the experimental unit was a single well of cells. For *in vivo* experiments, the experimental unit was an individual mouse.

No formal *a priori* sample size calculation was conducted. Sample sizes (e.g., n = 5 per group for *in vivo* experiments) were determined based on previous studies with comparable designs, feasibility constraints (time, cost, and animal availability), and the need to ensure sufficient biological and technical replicates for robust statistical analysis.

No *a priori* exclusion criteria were defined. All data points were included unless clear technical artefacts were identified. For zeta potential measurements of SREP4R, one replicate (−24.07 mV) was excluded as an outlier, identified using the median absolute deviation (MAD) method (modified Z-score >3.5). Outliers in the early endosome live cell imaging colocalisation assay were identified in GraphPad Prism using the ROUT method (Q = 5%), applied separately to each replicate curve.

For in vitro experiments each experimental group consisted of at least n = 3 independent biological replicates, each measured in technical triplicates. For *in vivo* experiments each experimental group consisted of at least n = 5 independent biological replicates.

Potential confounders such as treatment order or sample location were not formally randomised, but all groups were handled under the same experimental conditions to reduce bias. Group allocation was known to the experimenters during treatment and data collection. Outcome assessment and data analysis were performed with uniform software settings to reduce potential bias.

Multiple linear regression in R was performed using amino acid proportions (normalised), categorical modifications (“Stearyl,” “Acetyl,” “Cyclic”) encoded as factors, and min–max scaling, with AIC-based stepwise selection to refine the model. The final regression (incorporating specific residues, net charge, hydrophobicity, and structural modifications) explained 94.1% of the variance in encapsulation (adjusted R^2^ = 0.941). Simple linear regression and Pearson correlation (two-sided 95% CI) in GraphPad Prism were used to relate binding molar ratios to key quantitative properties. For motif discovery, the MEME suite was applied in classic mode to identify frequent siRNA-binding motifs and in discriminative mode to compare CPPs effective in both A375 and MCF7 cells against ineffective peptides, highlighting sequence features associated with functional delivery.

For internalisation studies, repeated-measures one-way ANOVA with Geisser–Greenhouse correction was followed by Šidák's multiple comparisons test. GAPDH silencing experiments were analysed by multiple paired t-tests, and *in vivo* data were compared with an unpaired t-test. Dose–response relationships were modelled by non-linear regression. Statistical significance was set at P < 0.05, with levels reported as ∗P < 0.05; ∗∗P < 0.01; ∗∗∗P < 0.001; ∗∗∗∗P < 0.0001. All statistical analyses were performed in GraphPad Prism [version X] unless otherwise stated.

Parametric tests were applied as implemented in GraphPad Prism, with built-in corrections where appropriate.

### Role of funders

The funders provided financial support only. They had no role in study design, data collection, data analysis, data interpretation, or writing of the report.

## Results

### Design of optimised DMBT1-derived peptides for siRNA encapsulation and delivery

**Fig. 1:**
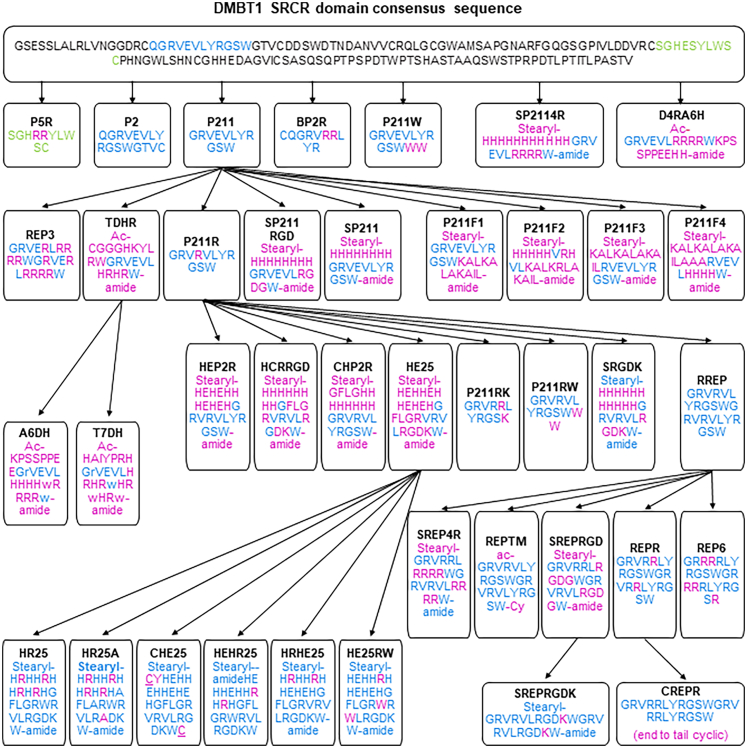
**Design of DMBT1-derived cell-penetrating peptides**. Schematic representation of the design process for DMBT1-derived cell-penetrating peptides, modelled on the consensus SRCR domain of DMBT1. Peptides derived from SRCRP1 are depicted in blue, and those derived from SRCRP5 are shown in green. Modifications introduced relative to earlier variants are highlighted in pink. Lowercase letters indicate d-amino acids, while underlined residues denote the positions of disulphide bridges.

The design strategy for the DMBT1-derived cell-penetrating peptides (DCPPs) builds on insights from our earlier work with SRCRP2-11 and SRCRP2-11R peptides.^28^ To enhance siRNA encapsulation efficiency, serum stability and cellular internalisation, we designed 37 peptides by systematically modifying key structural features of DMBT1-derived sequences (Fig. 1 and Supplementary Table S1).

Our approach utilised several strategies such as predictive CPP algorithms, incorporation of arginine residues, histidine tails, or hydrophobic amino acids, fusion between existing CPPs and targeting sequences, and introduction of chemical modifications. By adopting an “agile” project management method, we iteratively tested peptide sequences for CPP properties and refined them to develop improved versions.

Six peptides, including SRCRP2-11 (here named P211), were directly derived from the SRCR DMBT1 domain. For instance, P5R was designed by adding two arginine residues to the N-terminus of the SRCR DMBT1 domain, a region known to interact with HIV-1 gp120 and prevent viral infection, using the CellPPD predictive algorithm by Gautam et al.^34^^,^^35^

Additional peptides, such as P2, BP2R, P211W and SP2114R, were derived from P211 and modified with arginine, tryptophan, histidine, stearylation, and amidation to enhance positive charge, hydrophobicity, endosomal escape, siRNA interaction and serum stability.

Further modifications led to the creation of D4RA6H, designed from the DMBT1 sequence (GRVEVLXXXXW), with the “XXXX” motif replaced by an arginine-rich sequence (RRRR) for siRNA binding, and fused with a CD44-targeting moiety (KPSSPPEEHH) to increase tumour cell selectivity. Histidines at the C-terminus should facilitate endosomal escape by attracting protons and destabilising the endosomal membrane.

Additional derivatives were created from P211 by incorporating different functional groups. Peptides such as Rep3, SP211RGD and SP211 were developed by adding arginine, histidine and further chemical modifications. Peptides P211F1, P211F2, P211F3 and P211F4 were derived by fusion with the NickFect peptide, a CPP derived from Transportan (TP), itself originating from the neuropeptide galanin and the wasp venom peptide mastoparan. Similar to NickFect, these variants were stearylated at the N-terminus and amidated at the C-terminus. Peptides TDHR, T7DH and A6DH were created by combining P211 with transferrin or CD44 targeting moieties, further enhanced with d-amino acids, acetylation and amidation.^36^, ^37^, ^38^

We created peptides HEP2R, HCRRGD, CHP2R and HE25 from P211R, incorporating a pH-sensitive design with negatively charged N-terminal regions of histidine (H) and glutamic acid (E), masked by a complementary positively charged C-terminal.^39^ A cathepsin B-cleavable GFLG sequence was inserted between these domains to allow selective endosomal disassembly.^40^ Peptides HCRRGD and HE25 were further refined with an integrin-targeting RGD domain, and HE25 was further modified to produce arginine-rich and cyclic variants, (HR25, HR25A, CHE25, HEHR25, HRHE25, HE24RW) optimising delivery potential and bioactivity.

Additionally, peptides P211RK, P211RW and SRGDK, derived from P211R, were refined by adding arginine (R), tryptophan (W) or histidine (H) along with stearylation and amidation to enhance stability and cellular uptake. Finally, the peptide RREP was created from P211R through duplication of the sequence motif to improve its functional properties. Together, this led to variants with increased arginine content and modifications such as stearylation, amidation, acetylation and cyclisation.

### DCPPs–siRNA interaction and encapsulation are improved in the majority of the DCPP variants and favoured by hydrophobic residues and high charge density

**Fig. 2:**
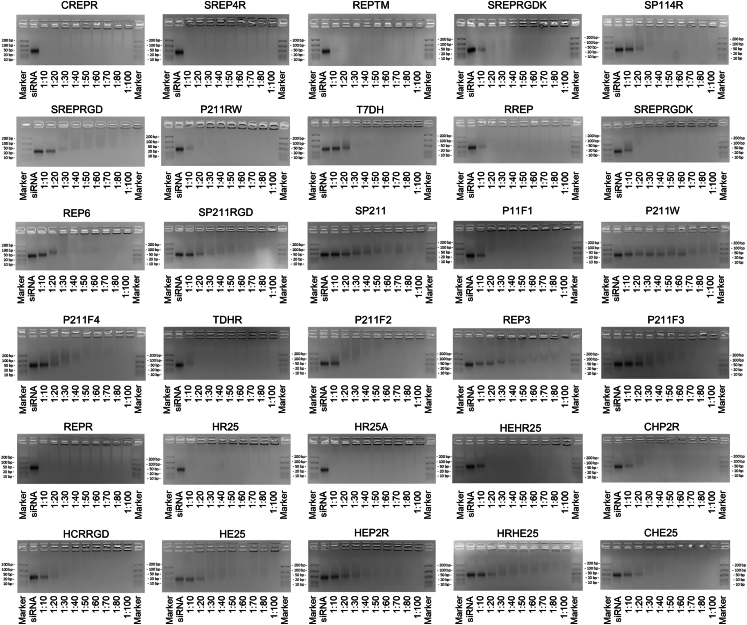
**Cell-penetrating peptide-mediated siRNA encapsulation**. Electrophoretic mobility shift assay demonstrating siRNA encapsulation by 30 DCPPs at varying binding molar ratios. Molecular marker band sizes are 10, 20, 50, 100 and 200 base pairs, providing size reference for DCPP–siRNA complexes.

**Fig. 3:**
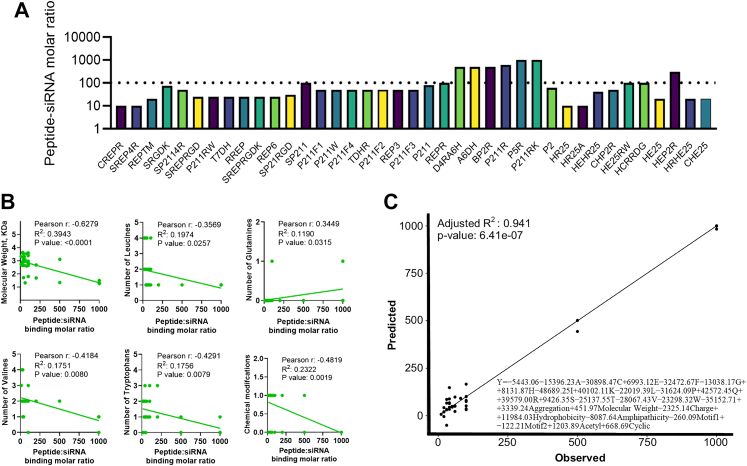
**Correlation between cell-penetrating peptide–siRNA binding molar ratios and peptide physicochemical properties**. (A) Binding molar ratios of siRNA to the 39 tested peptides. (B) Scatterplots showing Pearson's correlation and simple linear regression between selected peptide properties and binding molar ratios (n = 39; solid line represents the regression fit). (C) Comparison of predicted versus observed binding molar ratios from the multivariate regression model. The regression equation is displayed within the panel; full model details are provided in Supplementary Table S2.

**Supplementary Figure S1:**
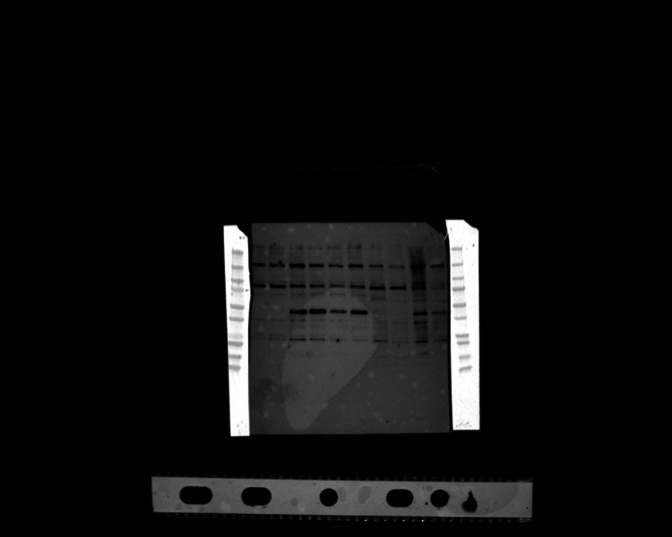


The peptide–siRNA binding molar ratio was evaluated using an electrophoretic mobility shift assay (EMSA). Thirty of the 37 DCPPs exhibited efficient siRNA encapsulation (low peptide:siRNA molar ratio), with 27 (73%) outperforming the previously designed P211 and P211-R in terms of encapsulation efficiency (Figs. 2 and 3A, and Supplementary Figure S1). Interestingly, both CD44-targeting peptides containing the A6 motif, A6DH (Ac-KPSSPPEEGrVEVLHHHHwRRRRw-NH_2_) and D4RA6H (Ac-GRVEVLRRRRWKPSSPPEEHH-NH_2_) showed no detectable siRNA binding (Figs. 2 and 3A, and Supplementary Figure S1). In contrast, most integrin-targeting peptides containing the RGD sequence, such as SREPRGD, SREPRGDK, SP211RGD, HCRRGD, and HE25, efficiently bound siRNA (Figs. 1–3A).

**Supplementary Figure S2:**
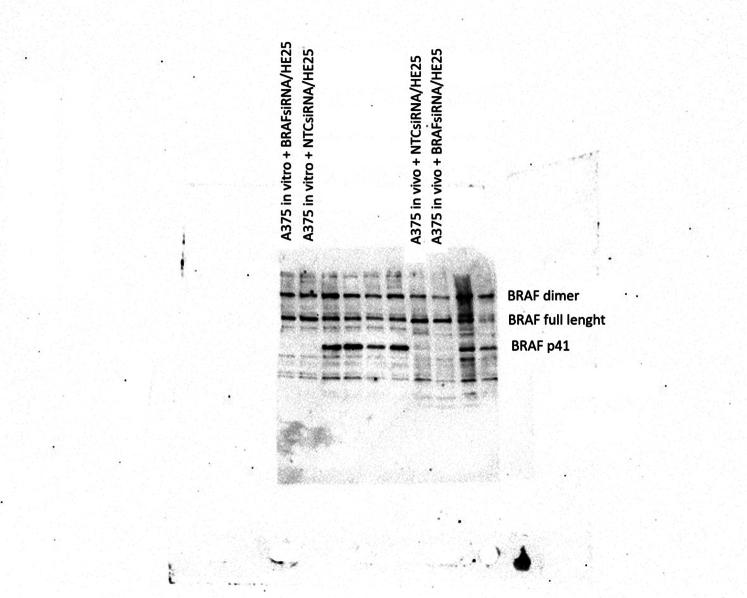


To identify the peptide properties influencing siRNA binding, we adopted an approach inspired by Hebditch et al., analysing amino acid composition along with 7 composite features: K-R, D-E, K + R, D + E, K + R-D-E, K + R + D + E and F + W + Y (Supplementary Table S1). Additionally, we examined 8 predicted peptide properties: aggregation propensity, molecular weight, net hydrogen, charge at pH 7, pI, hydrophobicity, hydrophilicity and chemical modifications (including d-amino acids, stearylation, amidation and cyclisation).^41^ We identified three distinct motifs in the peptide sequences by querying the MEME suite (Supplementary Figure S2).^42^ The first motif is enriched in arginine (R) and leucine (L), with a conserved tryptophan (W) at the C-terminus. These residues provide a balance of positive charge and hydrophobic/aromatic interactions that are well suited for nucleic acid binding. Notably, glycine (G) frequently occurs in this motif. While not basic or hydrophobic, glycine is the smallest amino acid and contributes structural flexibility, which may facilitate the adaptation of the peptide backbone to RNA surfaces or to multimeric peptide–RNA assemblies.^26^^,^^28^ The second motif shows strong conservation of histidine (H), arginine (R), lysine (K), and alanine (A). The combination of charged and small residues suggests potential for helical structural organisation, as well as electrostatic interactions with siRNA, features reminiscent of NickFect peptides.^36^

The third motif is characterised by repeats of histidine (H) interspersed with arginine (R), followed by glycine (G), phenylalanine (F), and leucine (L). The prevalence of histidine and arginine suggests a strong electrostatic component to binding, while the aromatic and hydrophobic residues at the C-terminus may stabilise peptide–RNA interactions through stacking or hydrophobic contacts.

The amino acid composition, composite features, predicted physicochemical properties and chemical modifications were correlated with the peptide-siRNA binding molar ratio. These relationships were analysed one-to-one individually using correlation analysis and collectively through a multiple linear regression model. The combined approach aimed to identify key factors influencing binding efficiency, with the regression model providing a more comprehensive view of how these diverse properties interact to affect the binding molar ratio, thus allowing better understanding of how structural and chemical variations in peptides influence their capacity to encapsulate siRNA effectively. When analysing the correlation between peptide–siRNA binding molar ratio and individual properties, we observed inverse correlations between the binding molar ratio and factors such as molecular weight, the presence of leucine, valine and tryptophan residues, as well as chemical modifications like acetylation, stearylation, or cyclic structures (Figs. 2 and 3B and Supplementary Table S3).

This suggests that these properties enhance binding efficiency by facilitating more compact and stable interactions between the peptides and siRNA, ultimately reducing the number of peptides needed for effective encapsulation. In contrast, glutamines increase the binding molar ratio, which negatively affects siRNA encapsulation efficiency by potentially reducing the strength or stability of peptide–siRNA interactions.

The multiple linear regression analysis of peptide properties demonstrates a strong model fit (R^2^ = 0.941, *p* = 6.41e-07), indicating that the chosen variables account for a significant portion of the variance in peptide–siRNA binding molar ratios (Fig. 3C and Supplementary Table S2). The analysis revealed that hydrophobic amino acids such as I, L, V and F, along with P and C, enhanced siRNA encapsulation by reducing the binding molar ratio and increasing the binding efficiency. Increased positive charge improved siRNA binding, likely through enhanced electrostatic interactions. Conversely, glutamine and arginine increased the binding molar ratio, possibly due to their influence on peptide structure or interaction dynamics, which may reduce binding efficiency. These results underscore the need to balance hydrophobicity and charge for optimal siRNA delivery.

### DCPP–siRNA stability in serum is enhanced in siRNA-interacting DCPPs and favoured by hydrophobic residues, increased charge and chemical modifications

**Fig. 4:**
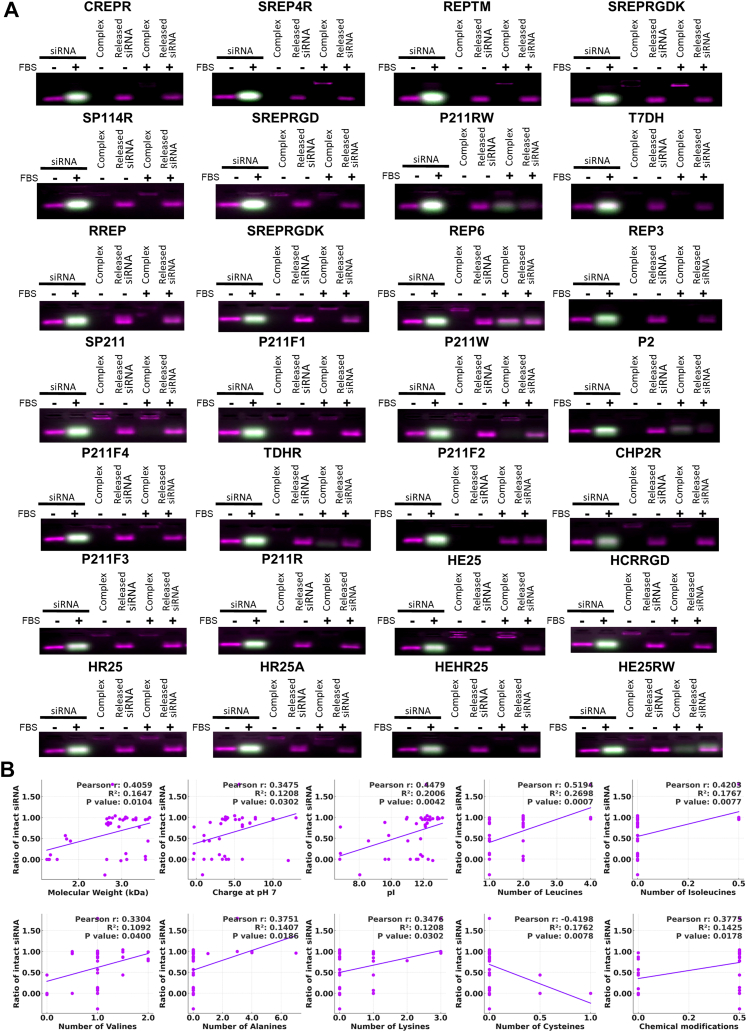
**Stability of cell-penetrating peptide–siRNA complexes in serum**. (A) FRET-based Electrophoretic Mobility Shift Assay (EMSA) evaluating the stability of the most stable DCPP-siRNA complexes at their binding molar ratios in 2.5% foetal bovine serum (FBS). Red lanes indicate intact siRNA, while green lanes denote siRNA degradation or band shifts due to peptide complexing and interaction. The stability of complexed and released FRET-siRNA is shown in the absence (−) and presence (+) of FBS. (B) Scatterplots showing Pearson's correlation and simple linear regression between selected peptide properties and stability in serum (n = 39; solid line represents the regression fit).

**Supplementary Figure S3:**
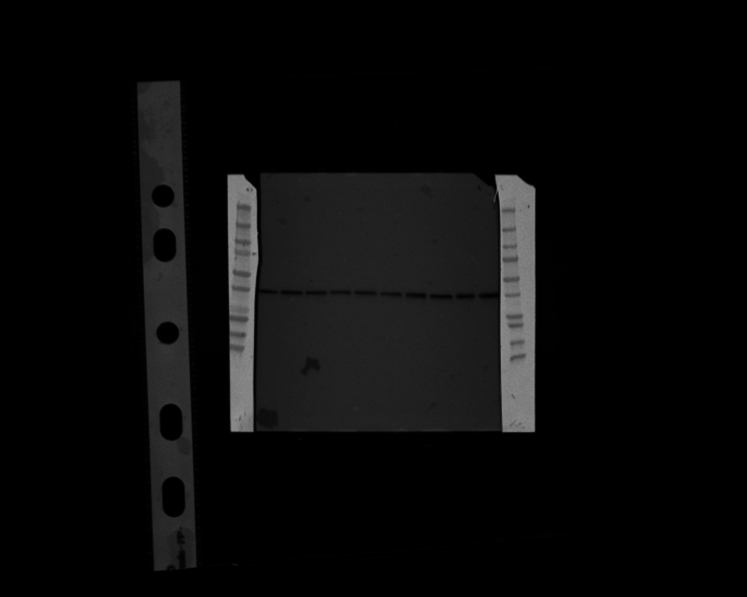


Next, the stability of the peptide–siRNA complexes was assessed using a FRET electrophoresis assay that we have developed.^32^^,^^33^ The 27 DCPP variants exhibiting enhanced encapsulation efficiency all also demonstrated enhanced stability in serum (Fig. 4A and Supplementary Figure S3). Correlation analysis between peptide properties and the ratio of intact siRNA revealed several key factors contributing to peptide–siRNA complex stability (Fig. 4B and Supplementary Table S3). Peptides with higher molecular weight and increased charge at pH 7 and pI demonstrated more robust protection of siRNA. Specific amino acids, such as alanine, cysteine, isoleucine, leucine, valine and lysine, positively influenced siRNA stability, likely due to their hydrophobic or electrostatic properties, which enhance peptide–siRNA binding. Additionally, chemical modifications, including acetylation and stearylation, further increased stability by promoting hydrophobic interactions and shielding the siRNA from degradation.

These results highlight the importance of optimising peptide physicochemical properties to improve the protective efficacy of peptide–siRNA complexes in serum.

### Optimised DCPPs exhibit enhanced cell interaction with MCF7 and A375 cells

**Fig. 5:**
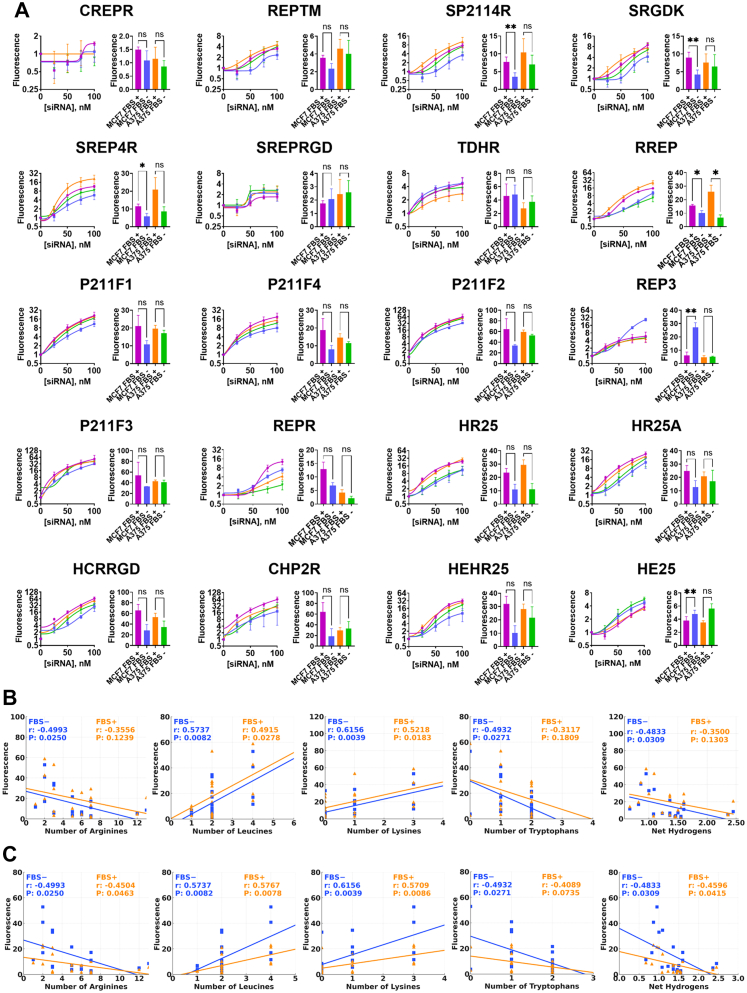
**Internalisation of cell-penetrating peptide–siRNA complexes in cancer cells**. (A) Internalisation of DCPP-siRNA complexes in A375 and MCF7 cells was assessed at varying siRNA concentrations (dose–response curve, left) and at a fixed 100 nM siRNA (bar graph, right). Data were analysed using GraphPad Prism, with dose–response curves from non-linear regression. Bars represent mean values with SD from three biological replicates. Statistical significance was determined using RM one-way ANOVA with Geisser–Greenhouse correction and Šidák's test (∗P < 0.05; ∗∗P < 0.01; ∗∗∗P < 0.001; ∗∗∗∗P < 0.0001). (B) Linear regression and Pearson's correlation analyses showing significant relationships between peptide properties and internalisation, with results for conditions with (orange) and without (blue) FBS. (n = 20; solid line represents the regression fit).

We investigated the interaction capacity of DCPP–siRNA complexes with cells and assessed the potential interference of FBS on these interactions. Fluorescent-labelled BLOCK-iT™ Alexa Fluor siRNA was encapsulated with the designed peptides and administered to MCF7 and A375 cells both in the presence and absence of FBS. Following washing, fluorescence intensity was measured for a detailed comparison of the complexes' binding and internalisation efficiency under different serum conditions (Fig. 5A).

Among the 27 DCPPs that were stable in the serum, 20 exhibited enhanced interaction compared to the previously designed variants interaction with A375 and MCF7 cells, with the interaction increasing proportional to the complex concentration. For some variants, including CREPR, SREPRGD, REP3 and REPR, a characteristic sigmoid curve with a plateau was observed, indicating the involvement of an active, saturable interaction mechanism, likely receptor mediated. For most variants, there was no significant difference in cell interaction between the presence and absence of FBS; however, for some variants, such as SP2114R, SRGDK and RREP, the presence of FBS enhanced cell interaction, while for other variants such as REP3 and HE25, reduced interaction with A375 cells was observed in the presence of FBS, suggesting interference under these conditions (Fig. 5A).

We also correlated cell internalisation to MCF7 and A375 cells in the presence and absence of FBS with various peptide properties (Fig. 5B and C, and Supplementary Table S3). The analysis revealed that the net hydrogen content and the number of arginines and tryptophans negatively impacted binding to both cell lines regardless of the presence of serum. This may be due to the guanidinium group of arginine, which forms stronger and more directional electrostatic and hydrogen-bonding interactions than lysine. Such interactions can lead to overly stable peptide–membrane or peptide–RNA contacts, reducing the dynamic binding needed for efficient uptake and potentially promoting peptide aggregation through multivalent crosslinking. Tryptophan, due to its strong affinity for the phospholipid interface, may stabilise peptide association with the membrane surface but does not necessarily promote translocation across the bilayer. In contrast, the number of lysines and leucines positively affected internalisation across both cell lines with or without serum. Lysine's positive charge likely contributes to favourable interactions that drive membrane translocation, while leucine, a hydrophobic residue, may support insertion into the lipid environment and stabilise peptide–membrane contacts, thereby promoting effective internalisation. Overall, most DCPP–siRNA complexes exhibited robust cell interactions independent of serum, with lysine and leucine favouring internalisation, whereas arginine, tryptophan, and net hydrogen content were associated with reduced binding efficiency.

### Sequence-specific motifs of siRNA-delivering peptides govern effective gene silencing and are independent of physicochemical properties

**Fig. 6:**
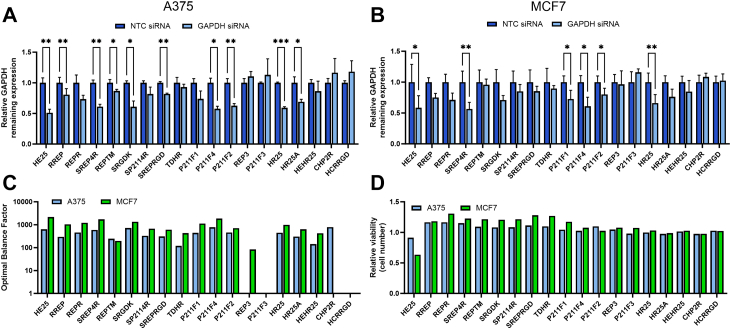
**GAPDH silencing in cancer cells using cell-penetrating peptide–GAPDH siRNA complexes**. Silencing of GAPDH in A375 (A) and MCF7 (B) cells by DCPP/GAPDH siRNA complexes. (C) Optimal balance factor and (D) relative viability (cell number) of A375 and MCF7 cells. Bars represent the mean values, with error bars indicating the standard deviation (SD) calculated from three biological replicates. Data were analysed using GraphPad Prism, employing multiple paired t-test. Significance levels are indicated as ∗P < 0.05; ∗∗P < 0.01; ∗∗∗P < 0.001; ∗∗∗∗P < 0.0001.

We evaluated the gene silencing potential of newly designed DCPP by complexing them with GAPDH-targeting siRNA and administering the DCPP/siRNA complexes to A375 and MCF7 cells. Several DCPP/siRNA complexes exhibited efficient GAPDH silencing in both A375 and MCF7 cell lines (Fig. 6A and B). In A375 cells, peptides such as HE25, RREP, SREP4R, REPTM, SRGDK, SREPRGD, P211F4, P211F2, HR25, and HR25A exhibited significant gene silencing. Peptides HE25, SREP4R, P211F1, P211F2, P211F4, and HR25 also demonstrated significant silencing effects in MCF7 cells, with HE25, SREP4R, and HR25 emerging as the most promising DCPP candidates across both cell lines. Notably, these peptides exhibited a high optimal balance factor (OBF), calculated as GAPDH expression level of NTC (non-targeting control) siRNA samples × % knockdown, indicating their effective silencing capacity. Furthermore, they maintained high cell viability and thus limited cytotoxicity, supporting their potential for therapeutic applications (Fig. 6C and D).

**Supplementary Figure S4:**
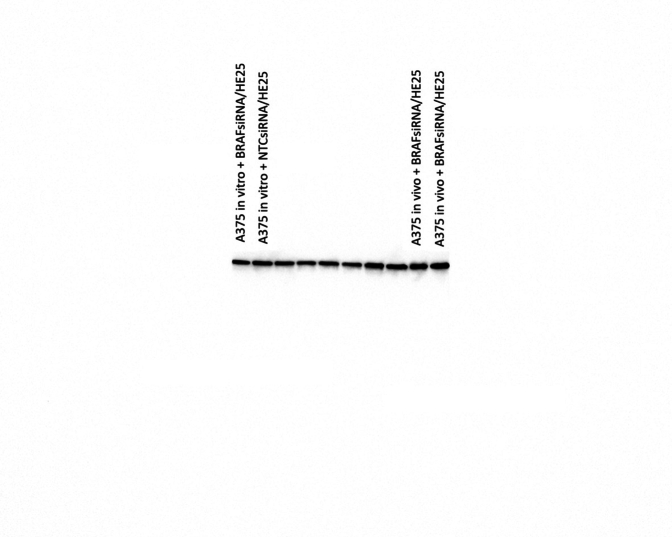


Given that only the peptides that passed rigorous criteria for siRNA encapsulation, serum stability and cell internalisation were tested for silencing, we anticipated these properties would contribute significantly to gene silencing. However, our correlation analysis found no clear relationship between silencing efficiency and individual peptide properties such as molecular weight, charge, or hydrophobicity (Supplementary Table S3), suggesting that once the fundamental criteria for siRNA delivery are met, these basic properties may no longer be the primary determinants of silencing efficiency. Furthermore, we performed a motif search using the discriminative mode of the MEME suite, which identifies motifs significantly different between two sets of sequences. This analysis revealed that the motif SWGRVRVLRGDKW is significantly more prevalent in the sequences of CPPs that effectively facilitated gene silencing, as validated in both cell lines (Supplementary Figure S4). The successful CPPs include HE25, RREP, REPR, SREP4R, SRGDK, P211F1, P211F4 and HR25. This motif aligns with the DMBT1 peptide sequence SRCRP2 from which most peptides are derived.^42^

Overall, our results indicate that for peptides capable of interacting with siRNA, stabilising it in serum, and engaging with cancer cells, the ultimate ability to facilitate gene silencing is not dependent on their physicochemical properties, but rather relies on specific sequence patterns.

### Mechanistic characterisation of siRNA/peptide complexes

To gain mechanistic insights into how the designed peptides function as cell-penetrating peptides (CPPs), we next characterised siRNA/peptide complexes using nanoparticle tracking analysis, zeta potential measurements, aggregation assays, and colocalisation studies with endosomal and lysosomal compartments in A375 cells. Based on their differential performance in the uptake and silencing assays, we selected HE25, SREP4R, and HR25 (top-performing CPPs) and TDHR, HCRRGD, and CHP2R (less efficient CPPs) for further investigation.

**Fig. 7:**
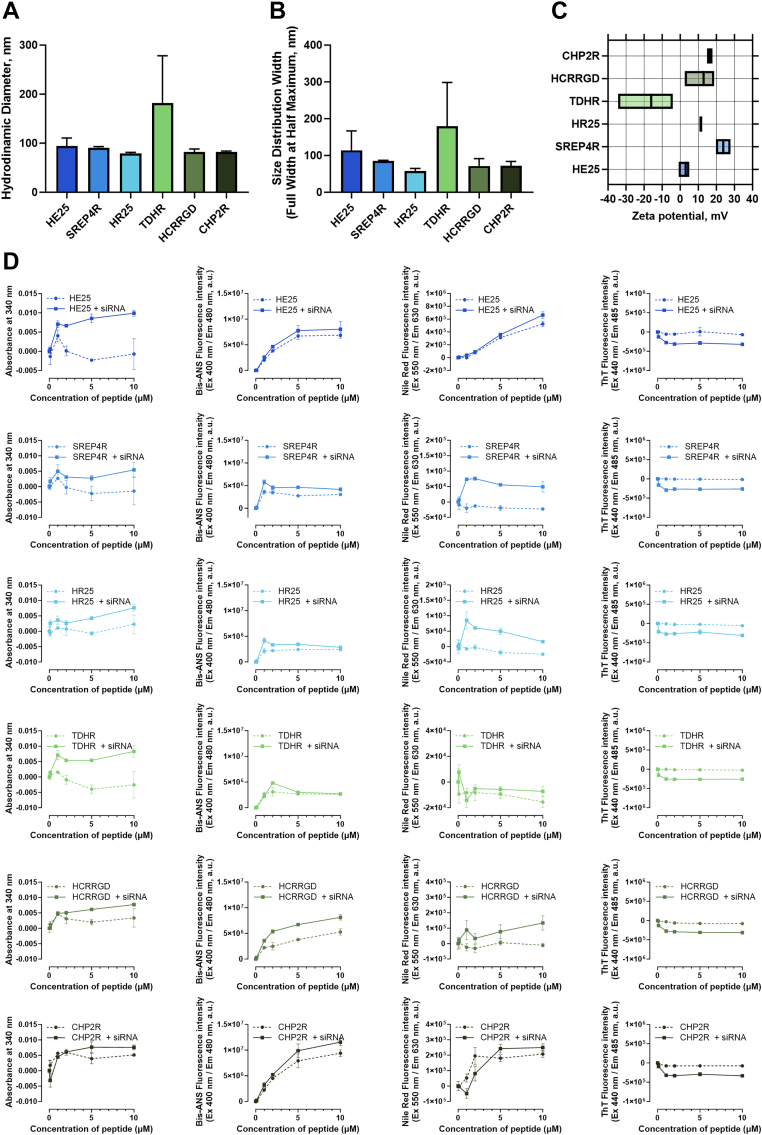
**Characterisation and aggregation assays of selected siRNA/peptide complexes**. (A) Hydrodynamic diameter, (B) size distribution (FWHM), and (C) zeta potential determined by nanoparticle tracking analysis (NTA). Data represent the mean of three independent batch replicates. (D) Aggregation assays of selected peptides assessed by absorbance at 340 nm (left panels), Bis-ANS fluorescence (middle left), Nile Red fluorescence (middle right), and Thioflavin T fluorescence (right), performed in the presence or absence of siRNA and at increasing peptide concentrations (n = 2).

Nanoparticle tracking analysis demonstrated that siRNA/peptide complexes formed particles predominantly in the 80–120 nm range, except for TDHR, which generated larger assemblies with a mean diameter around 180 nm and greater variability (Fig. 7A). Size distribution width (FWHM) was narrow for most complexes, whereas TDHR showed broader distributions, indicating higher polydispersity (Fig. 7B).

Zeta potential measurements revealed clear differences among peptides (Fig. 7C). SREP4R formed highly positively charged complexes (+20 to +30 mV), HCRRGD and CHP2R showed moderately positive charges (+10 to +20 mV), and HR25 was mildly positive (∼+10 mV). HE25 produced nearly neutral complexes, whereas TDHR carried a distinctly negative charge (−30 to −10 mV). These findings indicate that peptide sequence strongly dictates the surface charge of the resulting siRNA nanocomplexes (Fig. 7C). Notably, the near-neutral charge of HE25 may be advantageous by reducing nonspecific interactions with serum proteins and immune components, potentially supporting improved biocompatibility.

Aggregation assays revealed distinct peptide-dependent behaviours upon complexation with siRNA (Fig. 7D). Turbidity measurements (Abs 340 nm) showed only modest increases for most peptides, indicating limited formation of large aggregates, except for TDHR, which displayed a concentration-dependent rise suggestive of less stable assemblies. Bis-ANS fluorescence, reflecting exposure of hydrophobic regions, was strongly increased for HE25, SREP4R, and HCRRGD in the presence of siRNA, indicating the formation of ordered hydrophobic interfaces. Nile Red staining further supported micelle-like or amphipathic aggregate formation for HE25 and SREP4R, whereas HR25 and TDHR showed minimal signal, consistent with reduced ability to form amphipathic aggregates. Thioflavin T assays did not reveal significant β-sheet–rich aggregates for any peptide, suggesting that the complexes do not adopt amyloid-like structures. Taken together, these results indicate that efficient CPP-like peptides (HE25, SREP4R) tend to form hydrophobic and micelle-like assemblies with siRNA, whereas less effective peptides (TDHR, CHP2R) form heterogeneous or unstable aggregates.

Notably, HE25 and SREP4R displayed self-assembly tendencies even in the absence of siRNA, while HR25, TDHR, HCRRGD, and CHP2R required siRNA complexation to trigger detectable aggregation.

**Fig. 8:**
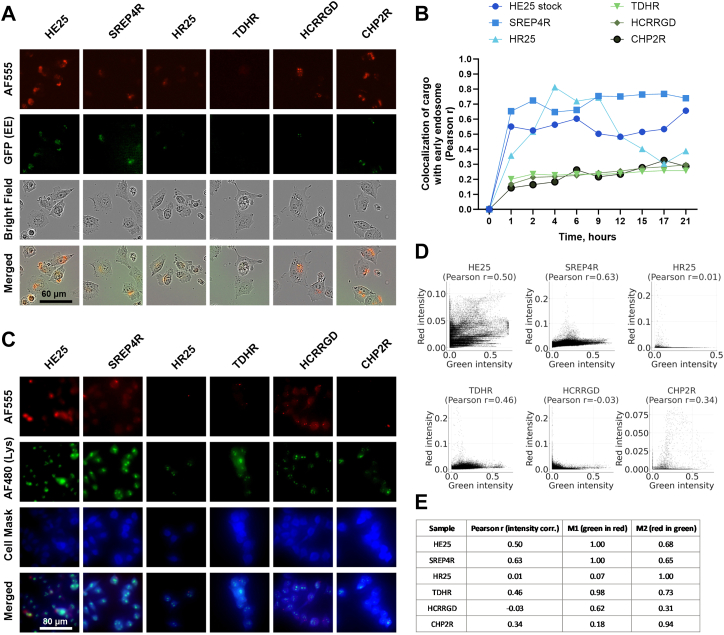
**Colocalisation of siRNA/peptide complexes with endosomal and lysosomal compartments**. (A) Fluorescent live-cell imaging (IncuCyte) of Alexa Fluor 555-labelled siRNA/peptide complexes (pseudocoloured red) in A375 cells, showing colocalisation with GFP-labelled early endosomes (BacMam marker, pseudocoloured green) at 3 h. Bright-field and merged channels are included. (B) Real-time live-cell tracking of colocalisation between GFP-labelled early endosomes (green) and Alexa Fluor 555-labelled siRNA (red). Corresponding videos are provided as Supplementary Information (Videos S1-6). (C) Colocalisation of siRNA/peptide complexes (Alexa Fluor 555, pseudocoloured red) with lysosomes, visualised using LAMP1–Alexa Fluor 480 (pseudocoloured green), after 24 h. Cell Mask was used to stain the cell cytoplasm and nuclei (pseudocoloured blue). Merged channels are included. (D) Colocalisation scatterplots and Pearson's correlation coefficients corresponding to the samples shown in (C). (E) Table summarising Pearson's and Manders' colocalisation coefficients for the samples shown in (C).

Live-cell colocalisation analysis of Alexa Fluor 555-labelled siRNA/peptide complexes with GFP-tagged early endosomes in A375 cells revealed marked differences among peptides (Fig. 8A–B and Videos 1–6). Efficient CPP-like peptides (HE25, SREP4R, HR25) displayed rapid and sustained endosomal association, reaching Pearson's correlation coefficients of 0.6–0.8 within the first 2–4 h and remaining stable over 21 h. In contrast, less effective peptides (TDHR, HCRRGD, CHP2R) showed only minimal colocalisation, plateauing at r values around 0.2–0.3. These results indicate that HE25, SREP4R, and HR25 facilitate efficient siRNA internalisation through endosomal trafficking, while TDHR, HCRRGD, and CHP2R exhibit poor uptake and limited endosomal association.

Colocalisation of siRNA/peptide complexes with lysosomes was assessed 24 h post-transfection using LAMP1 staining (Fig. 8C–E). Fluorescent imaging revealed that HE25 and SREP4R showed strong AF555-siRNA signal and visible overlap with LAMP1-positive lysosomes, whereas weaker CPPs (HCRRGD, CHP2R) displayed little siRNA signal overall (Fig. 8C). Quantitative analysis confirmed this trend, with the highest Pearson's correlation coefficients for SREP4R (r = 0.63) and HE25 (r = 0.50), moderate for TDHR (r = 0.46), and negligible for HR25 and HCRRGD (Fig. 8D–E). Importantly, the higher lysosomal signal observed for HE25 and SREP4R may reflect their greater overall uptake, with a substantial fraction of siRNA trafficked into degradative compartments while another fraction potentially escapes to the cytosol. In contrast, weak CPPs might show low lysosomal colocalisation simply because they deliver very little siRNA to the cell in the first place. Thus, although lysosomal accumulation is detectable for the most efficient CPPs, their superior functional activity suggests, albeit indirectly, that they promote both high uptake and at least partial endosomal escape.

### HE25 peptide-delivered BRAF^V600E^ siRNA reduces A375 melanoma cell proliferation *in vitro* and suppresses xenograft tumour growth *in vivo*

Transfection of A375 cells with HE25 DCPP-delivered BRAF^V600E^ siRNA exhibited a significant reduction in cell proliferation *in vitro* (Fig. 9A and Supplementary Figure S6). Transfection of immortalised melanocytes (MelST), which lack the BRAF^V600E^ mutation, with BRAF^V600E^ siRNA did not alter cell proliferation. Western blotting confirmed BRAF^V600E^ silencing (Fig. 9C and Supplementary Figure S7). To evaluate the *in vivo* efficacy, female CIEA-NOG mice, a severely immunodeficient strain that permits engraftment of human tumour cells, mice were subcutaneously engrafted with A375 melanoma cells harbouring the BRAF^V600E^ mutation and subsequently treated with either BRAF^V600E^-targeting siRNA or a non-targeting siRNA control (Fig. 9B–E and Figure S5). Tumour volumes were measured every 2 days over a 10-day period using a callipre (n = 5 per group). Tumours of mice treated with BRAF^V600E^ siRNA showed a reduction in BRAF expression (Fig. 9C) and a significant reduction in growth compared to mice treated with a non-targeting siRNA control (Fig. 9B, D, and E), indicating that HE25 peptide successfully delivered BRAF^V600E^ siRNA *in vivo* and resulted in inhibition of tumour growth. The tumour growth inhibition calculated as TGI (%) = [1—(Mean Tumour Volume in Treatment Group/Mean Tumour Volume in Control Group)] × 100% was 56%. BALB/c mice were selected to evaluate the safety of the siRNA/HE25 complex, as this immunocompetent strain enables the assessment of acute immune responses and potential complement activation. The intravenous administration of the siRNA/HE25 complex did not result in any significant change in body weight of the mice over a 15-day period receiving bi-weekly treatments (Fig. 9F). Ki-67 immunohistochemistry was used to assess tumour cell proliferation following treatment. Tumours from mice treated with BRAF^V600E^ siRNA/HE25 displayed markedly reduced Ki-67 staining compared with those receiving NTC siRNA/HE25 (Fig. 9G). Quantification confirmed a significant decrease in the proportion of Ki-67-positive cells in the BRAF^V600E^ siRNA/HE25 group (Fig. 9H), consistent with reduced proliferative activity.

The findings are relevant to human biology, as CPPs were derived from a human protein and tested with human BRAF^V600E^ siRNA in xenografted melanoma cells. Generalisation is limited by the use of immunodeficient mice, though biosafety in BALB/c mice supports translational potential.

## Discussion

This study presents a systematic strategy for the design and optimisation of DMBT1-derived CPPs for siRNA delivery, addressing major challenges of stability and efficacy in serum-containing environments. By employing rational design principles and correlation techniques, we identified several promising peptides, with HE25 emerging as the most effective candidate for siRNA delivery *in vitro* and *in vivo*.

The systematic design and testing of 37 peptides led to significant advancements in siRNA encapsulation, serum stability, and biological effectiveness compared to earlier DMBT1-derived peptides.^31^ Enhanced siRNA encapsulation efficiency correlated with hydrophobic residues and increased charge density, aligning with studies highlighting the role of electrostatic and hydrophobic interactions in stabilising peptide–nucleic acid complexes.^43^^,^^44^ The negative correlation between molecular weight and hydrophobic residues (e.g., leucine and valine) with the binding molar ratio, coupled with their positive correlation with stability, reinforces the concept that compact, hydrophobic peptides are more effective in enhancing siRNA interaction and stability.^45^

Positively-charged residues like lysine enhanced electrostatic binding to siRNA, while hydrophobic residues (e.g., alanine, leucine) promoted stability by shielding siRNA from degradation. These findings align with studies showing that charge and hydrophobicity both enhance siRNA stabilisation. Lysine-rich peptides strengthen electrostatic bonds with siRNA,^46^ while hydrophobic modifications like stearylation boost degradation resistance by mimicking lipid structures.^47^ Balancing these properties is key for efficient siRNA delivery.

When the CD44-targeting A6 motif was incorporated into DMBT1-derived CPPs, the resulting constructs (A6DH and D4RA6H) lost their ability to bind siRNA and were excluded from further analysis. This suggests that while A6 has been reported to enhance tumour selectivity via CD44 interaction,^48^ its integration into CPP backbones may interfere with the structural requirements for siRNA complexation. In contrast, incorporation of RGD motifs preserved siRNA binding, consistent with previous studies where RGD-containing peptides supported both integrin targeting and nucleic acid delivery.^49^

The 20 peptides that exhibited robust cell interaction in both A375 and MCF7 cell lines, both in the presence and absence of serum, represent a significant advancement over previous CPPs, which often displayed reduced efficacy in serum-containing environments.^50^ This improvement highlights the importance of balancing positive charge and hydrophobicity for serum stability and cell interaction. The interplay between these properties may mitigate peptide aggregation, as seen with highly charged arginine-rich peptides, and enhance membrane penetration, consistent with findings from studies of NickFect peptides.^51^ These insights underscore the value of rational peptide design informed by both empirical testing and established principles in CPP research.

Despite the significant improvements observed, our findings revealed a lack of correlation between basic peptide properties and silencing efficiency. This disconnect may be attributed to factors beyond the initial peptide design, such as intracellular siRNA release, endosomal escape, and RISC loading efficiency. While peptides like HE25, SREP4R and P211F4 showed robust gene silencing, others with similar encapsulation and stability produced variable results, underscoring the complexity of delivery mechanisms and peptide-specific roles in siRNA uptake and activation.

Motif analysis offered additional insights beyond general physicochemical traits. The motif *SWGRVRVLRGDKW* was significantly overrepresented among CPPs that achieved consistent gene silencing, suggesting that sequence-specific patterns may facilitate intracellular trafficking and siRNA release even when overall charge and hydrophobicity are comparable. This aligns with studies of NickFect and other multifunctional CPPs, where conserved motifs promote endosomal escape and siRNA activation.^36^, ^37^, ^38^^,^^43^^,^^44^^,^^46^^,^^47^^,^^51^ These results highlight the value of combining physicochemical optimisation with motif-guided design to enhance functional delivery.

Mechanistic characterisation was carried out for six peptides, including three high-performing and three lower-performing candidates. Nanoparticle tracking analysis (NTA) showed that most CPP–siRNA complexes formed stable nanoparticles in the 80–120 nm range, consistent with sizes favourable for endocytic uptake. In contrast, less efficient peptides produced larger or more polydisperse assemblies, which are typically associated with reduced delivery efficiency.^52^ These findings support previous reports highlighting the critical role of nanoscale organisation in determining the stability and biological performance of nucleic acid delivery systems.^52^

Zeta potential analysis further highlighted how sequence influences the surface properties of the nanocomplexes. While several peptides formed positively or negatively charged complexes, HE25 produced particles with a near-neutral charge. This property is of potential interest, as near-neutral complexes are less likely to engage in nonspecific interactions with serum proteins and immune components, which may translate into improved biocompatibility *in vivo*.^53^ However, we acknowledge that this remains speculative and requires direct immunogenicity testing in future studies.

Peptide self-assembly into defined supramolecular nanostructures, such as micelles or amphipathic assemblies, has been increasingly recognised as a critical determinant of delivery efficacy in nucleic acid therapeutics. For instance, amphipathic peptide C6M1 was shown to form stable micelle-like assemblies with siRNA, significantly enhancing complex stability and serum protection across various buffer conditions.^54^ More broadly, peptide amphiphiles and other self-assembling systems have been leveraged to create nanostructures that facilitate protective encapsulation and controlled delivery of nucleic acids.^55^ In this light, our findings that HE25 and SREP4R form hydrophobic, ordered assemblies even without siRNA, while other peptides require siRNA to drive aggregation highlight the relevance of self-assembly as a beneficial design feature. That said, the distinction between functional self-assembly and non-productive aggregation remains critical for CPP design and warrants thorough future structural studies.

Peptide HE25, previously validated for siRNA delivery against SARS-CoV-2,^31^ also mediated BRAF^V600E^ silencing in melanoma models under serum conditions, resulting in significant tumour growth suppression *in vivo*. Together with SREP4R and related candidates, these results demonstrate that DMBT1-derived CPPs can function as stable, efficient, and modular carriers for siRNA. The ability to integrate tumour-targeting ligands or functional motifs underscores their potential as customisable precision therapeutics. Future efforts should prioritise improving endosomal escape and RISC loading, supported by structural and imaging studies, and integrating computational design approaches. This systematic motif-guided optimisation of DMBT1-derived CPPs with demonstrated *in vivo* efficacy, positions DCPPs as versatile platforms for nucleic acid therapeutics.

In this study, siRNA/HE25 complexes showed no effect on the viability of non-tumorigenic MelST melanocytes and did not cause weight loss in treated mice, supporting selectivity and tolerability; moreover, our previous work further demonstrated absence of systemic toxicity or lung pathology upon systemic administration.^31^

Our motif-guided optimisation of CPP physicochemical traits offers a general framework for siRNA delivery beyond melanoma, adaptable with targeting ligands for various cancers or other diseases. With enhanced serum stability and cellular uptake, these CPPs are promising vectors for nucleic acid therapeutics. Future work should focus on improving endosomal escape and RISC loading, using structural and imaging studies to unravel silencing mechanisms, and leveraging computational modelling or machine learning to accelerate peptide design. This strategy positions CPPs as versatile, customisable carriers for targeted therapies.

### Strengths and limitations

Strengths of this study include the systematic design and testing of 37 CPPs, the integration of computational analyses with mechanistic assays, and validation across cell lines and *in vivo* models, which enhances translational relevance. Limitations include the incomplete understanding of endosomal escape and RISC loading, and the fact that the potential advantage of HE25's near-neutral zeta potential remains hypothetical, as no direct immunogenicity or serum protein-binding assays were performed. Broader toxicology and structural studies will also be required to confirm safety and mechanism.

## Contributors

Martina Tuttolomondo led the study design and execution: she was the lead for data curation, formal analysis, investigation, methodology development, validation, visualisation, and project administration; she also took primary responsibility for writing the original draft and for review & editing, and equally contributed to funding acquisition. Mikkel Green Terp provided supporting contributions to methodology and assisted with supervision throughout the project. Stefan Vogel and Nazmie Kalisi contributed by designing and conducting the Nano Tracking Analysis and zeta potential experiments. Henrik Jørn Ditzel equally contributed to funding acquisition and project administration, led overall supervision, and shared equally in drafting the original manuscript as well as its review and editing. Martina Tuttolomondo, Mikkel Green Terp, and Henrik Ditzel accessed and verified the underlying data. All authors read and approved the final version of the manuscript.

## Data sharing statement

Data reported in this paper and any additional information will be shared upon request. Requests should be sent to the corresponding author Martina Tuttolomondo, mtuttolomondo@health.sdu.dk.

## Declaration of interests

The authors declare no conflict of interest.

## Appendix A Supplementary data

Video 1 Fluorescent live-cell imaging (Incucyte) of Alexa Fluor 555-labelled siRNA encapsulated with HE25 peptide (pseudocoloured red) in A375 cells, showing colocalisation with GFP-labelled early endosomes (BacMam marker, pseudocoloured green). Only the merged fluorescent and bright field channels are shown. Time-lapse images were captured every 30 min.

Video 2 Fluorescent live-cell imaging (Incucyte) of Alexa Fluor 555-labelled siRNA encapsulated with SREP4R peptide (pseudocoloured red) in A375 cells, showing colocalisation with GFP-labelled early endosomes (BacMam marker, pseudocoloured green). Only the merged fluorescent and bright field channels are shown. Time-lapse images were captured every 30 min.

Video 3 Fluorescent live-cell imaging (Incucyte) of Alexa Fluor 555-labelled siRNA encapsulated with HR25 peptide (pseudocoloured red) in A375 cells, showing colocalisation with GFP-labelled early endosomes (BacMam marker, pseudocoloured green). Only the merged fluorescent and bright field channels are shown. Time-lapse images were captured every 30 min.

Video 4 Fluorescent live-cell imaging (Incucyte) of Alexa Fluor 555-labelled siRNA encapsulated with TDHR peptide (pseudocoloured red) in A375 cells, showing colocalisation with GFP-labelled early endosomes (BacMam marker, pseudocoloured green). Only the merged fluorescent and bright field channels are shown. Time-lapse images were captured every 30 minutes.

Video 5 . Fluorescent live-cell imaging (Incucyte) of Alexa Fluor 555-labelled siRNA encapsulated with HCRRGD peptide (pseudocoloured red) in A375 cells, showing colocalisation with GFP-labelled early endosomes (BacMam marker, pseudocoloured green). Only the merged fluorescent channels are shown. Only the merged fluorescent and bright field channels are shown. Time-lapse images were captured every 30 min.

Video 6 Fluorescent live-cell imaging (Incucyte) of Alexa Fluor 555-labelled siRNA encapsulated with CHP2R peptide (pseudocoloured red) in A375 cells, showing colocalisation with GFP-labelled early endosomes (BacMam marker, pseudocoloured green). Only the merged fluorescent and bright field channels are shown. Time-lapse images were captured every 30 min.
