# Supplementary material for: Systematic design of DMBT1-derived peptides correlating physicochemical properties and sequence motifs with siRNA delivery and efficacy in cancer therapy
Source: eBioMedicine. 2025 Oct 22;121:105977. doi: 10.1016/j.ebiom.2025.105977 (PMC12590273; doi:10.1016/j.ebiom.2025.105977)
Supplement: Supplementary Tables and Figures [file mmc7.docx]

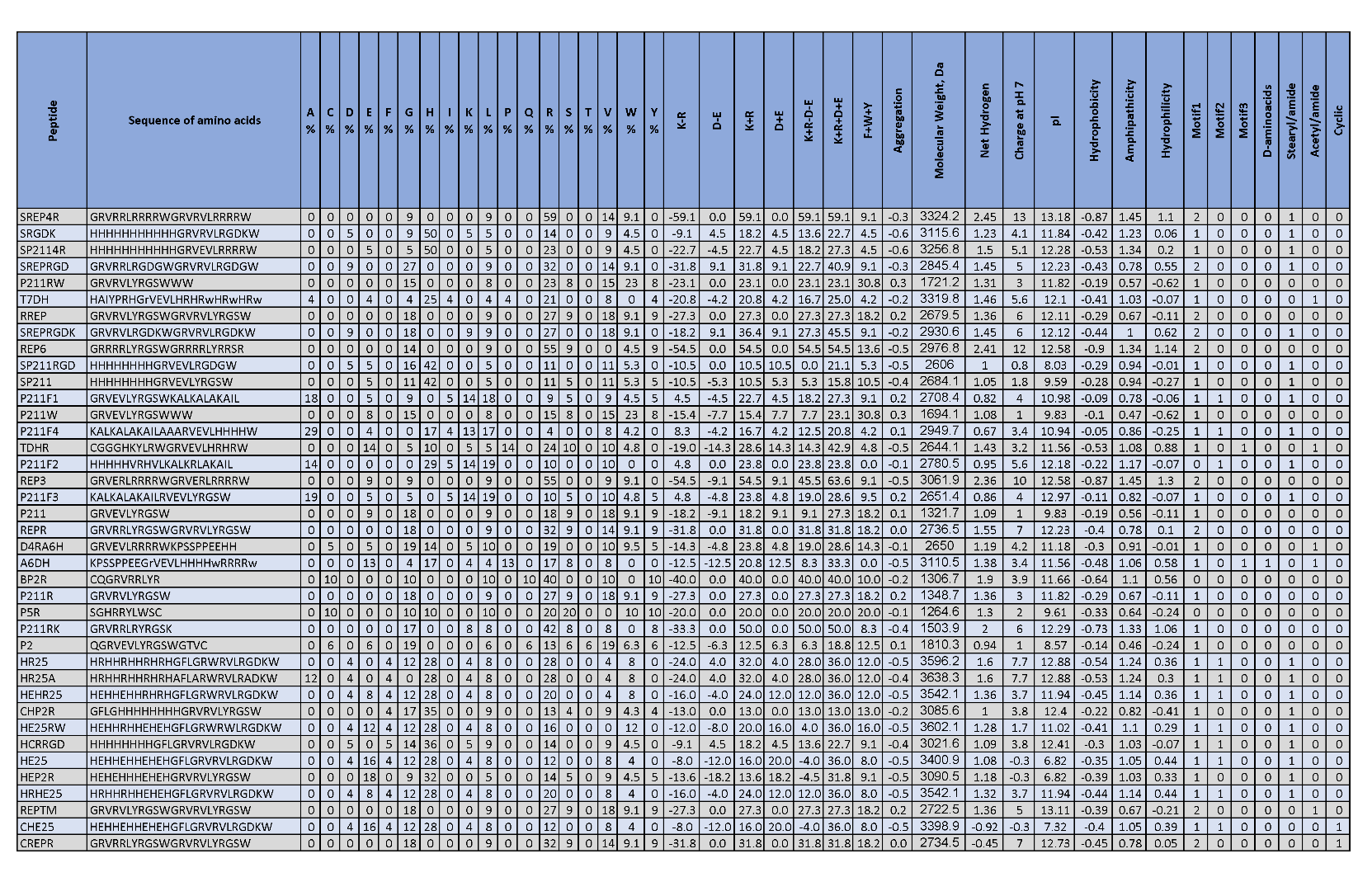


**Table S1.** Name, sequences and calculated properties of DCPPs.


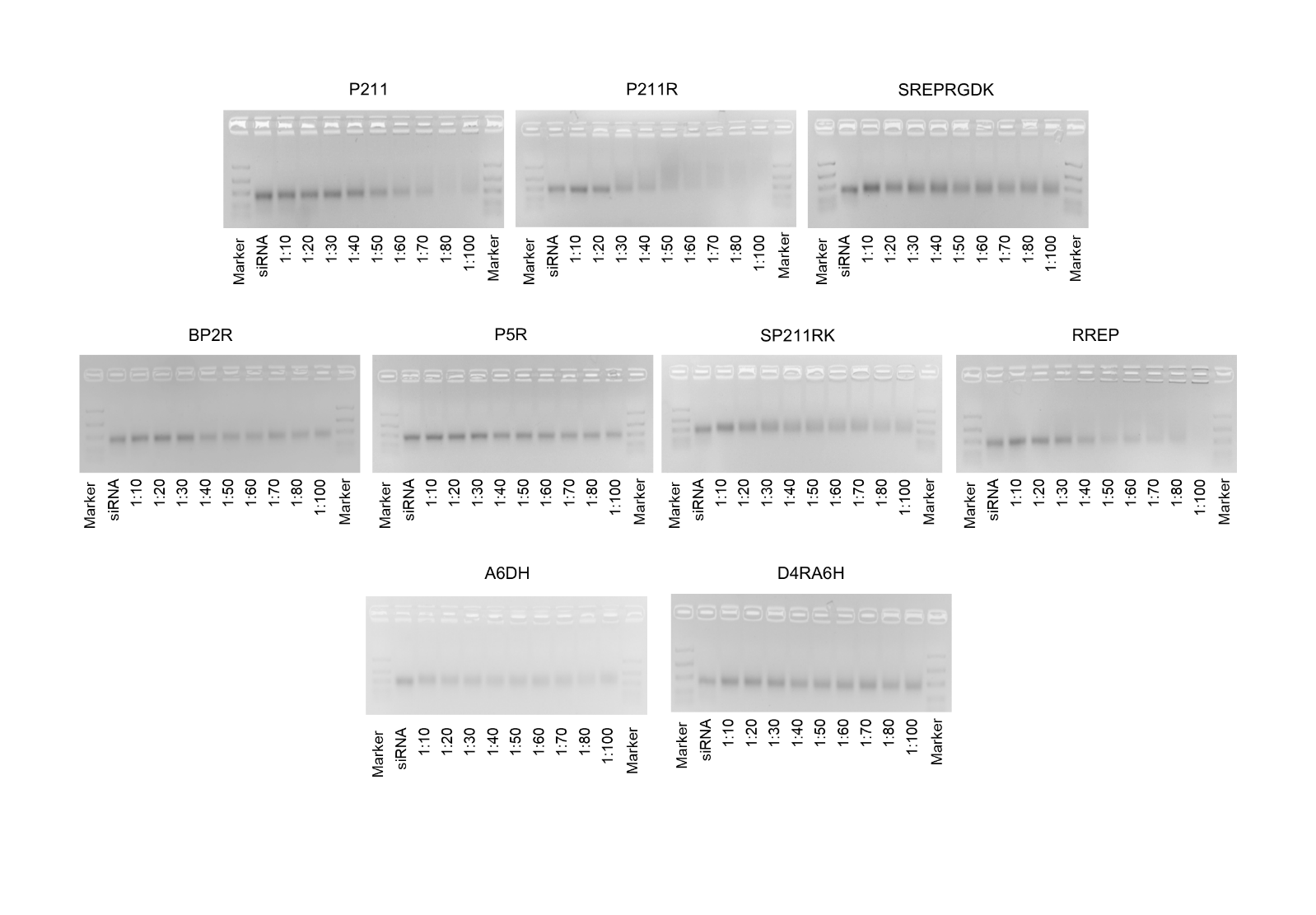


**Figure S1.** Electrophoretic Mobility Shift Assay (EMSA) showing peptides that failed to bind siRNA.


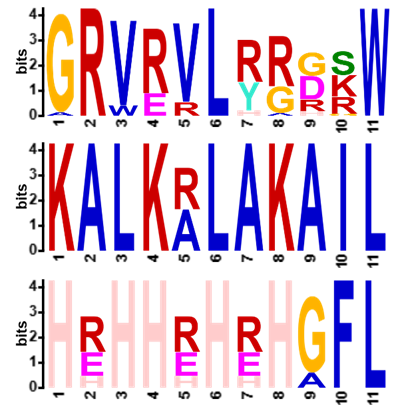


**Figure S2.** Motifs driving siRNA binding, identified using MEME Suite. Statistical analysis included log-likelihood ratios, E-values, and a background model to assess motif significance. All three motifs exhibited P-values below 0.0001, indicating high statistical significance.


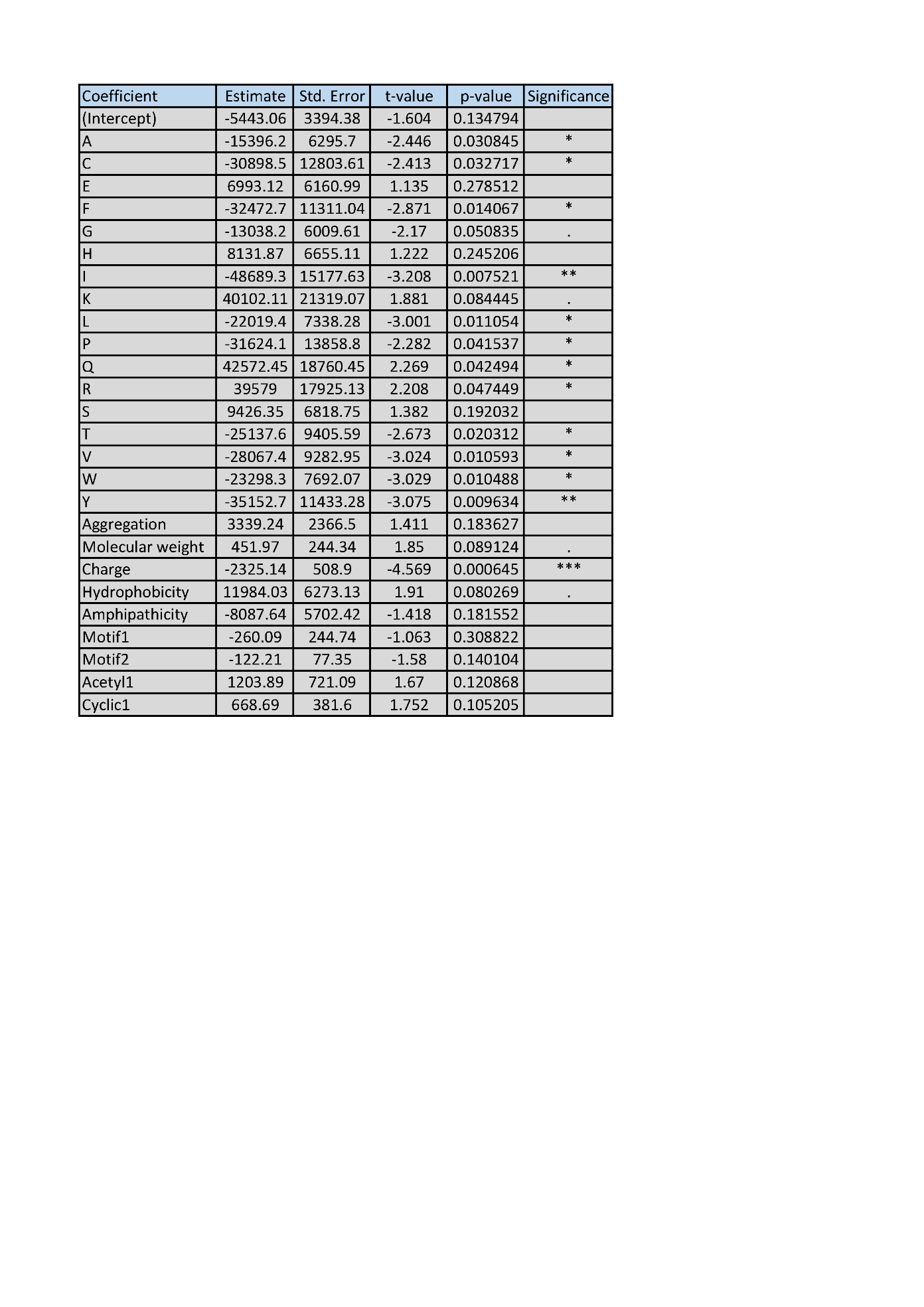


**Table S2.** Multiple linear regression model analyzing the relationship between DCPP-siRNA encapsulation efficiency and physicochemical properties. Statistical significance is indicated as follows: .P <0.1; *P < 0.05; **P < 0.01; ***P < 0.001; ***P < 0.0001.





**Table S3.** Pearson correlation analysis of the relationships between DCPP-siRNA encapsulation efficiency, stability in FBS, cellular internalization, and silencing facilitation with the physicochemical properties of DCPPs.


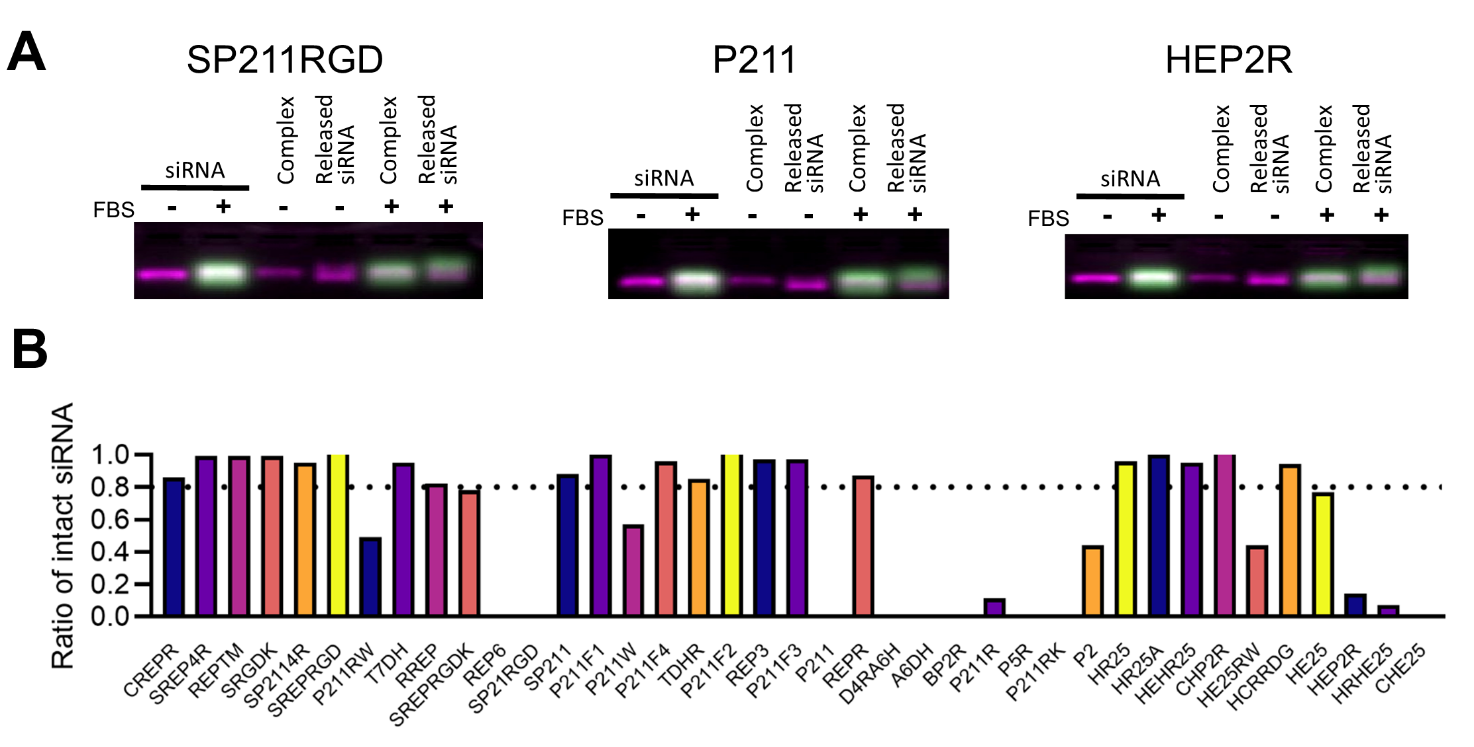


**Figure S3**. (A) EMSA-FRET assay evaluating siRNA stability in FBS, highlighting peptides that failed to protect FRET-labeled siRNA. (B) Calculated ratios of intact siRNA for each DCPP-FRET siRNA complex, based on residual FRET efficiency after incubation in FBS.


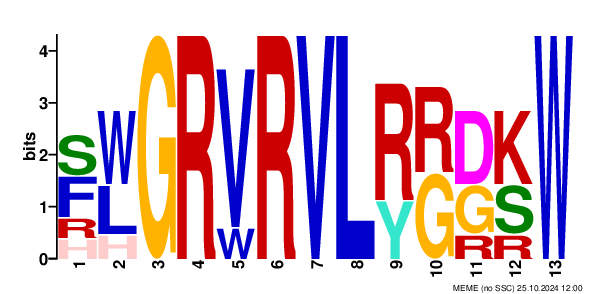


**Figure S4**. Motif driving gene silencing, identified using MEME Suite. Statistical analysis included log-likelihood ratios, E-values, and a background model to assess motif significance. The identified motif exhibited E-values below 0.0001 in DCPPs facilitating GAPDH silencing in both A375 and MCF7 cell lines.


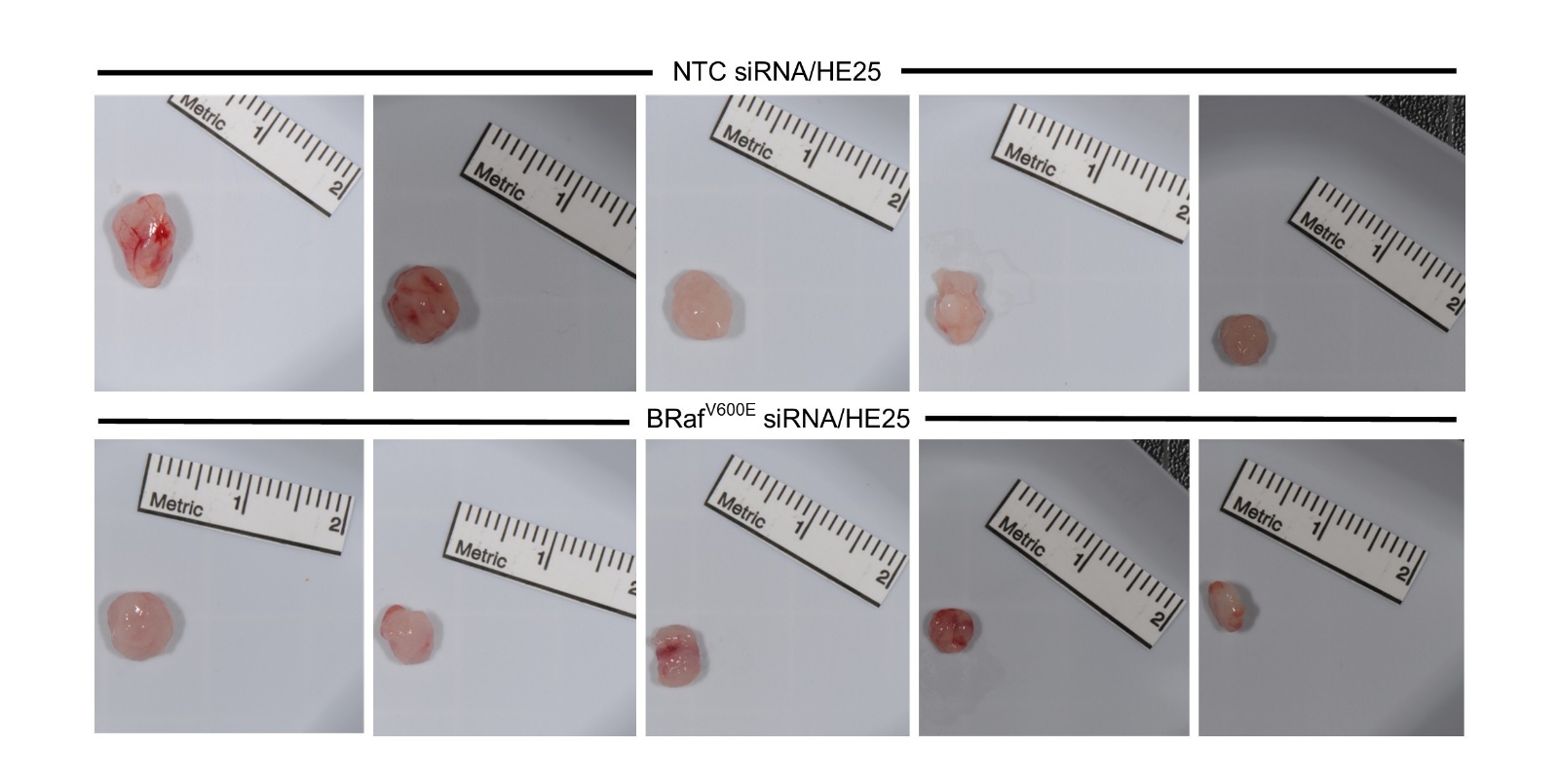


**Figure S5**. Photographs of excised tumors from the study groups corresponding to those presented in Figure 9B, with rulers included to indicate scale.


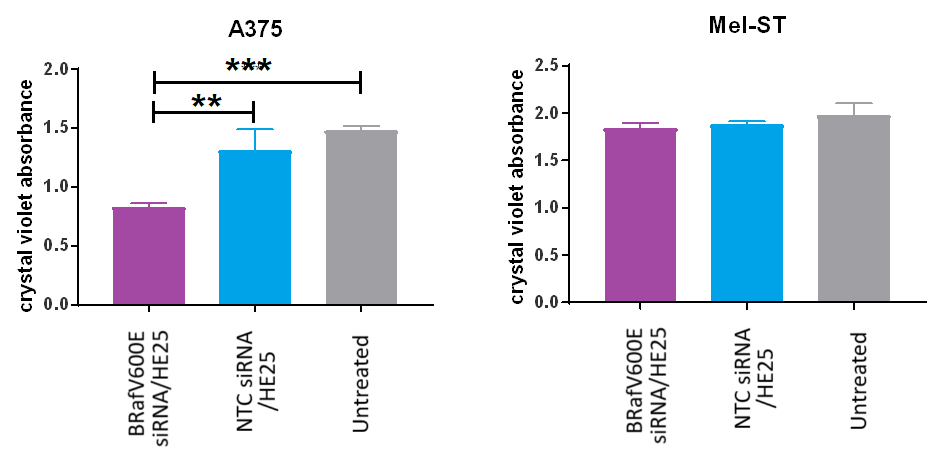


**Figure S6**. Cytotoxic effects of *BRaf*^V600E^ silencing *in vitro* following administration of *BRaf*^V600E^ siRNA/HE25 complexes in A375 melanoma cells and immortalized MelST melanocytes.


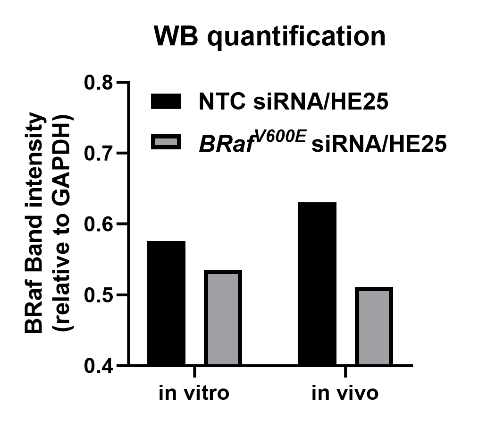


**Figure S7**. Densitometric quantification of BRAF protein bands detected in the Western blot. Intensities were normalized to loading controls and are shown relative to control samples.
